# TKI-addicted ROS1-rearranged cells are destined to survival or death by the intensity of ROS1 kinase activity

**DOI:** 10.1038/s41598-017-05736-9

**Published:** 2017-07-17

**Authors:** Hayato Ogura, Yuka Nagatake-Kobayashi, Jun Adachi, Takeshi Tomonaga, Naoya Fujita, Ryohei Katayama

**Affiliations:** 1 Division of Experimental Chemotherapy, Cancer Chemotherapy Center, Japanese Foundation for Cancer Research, Tokyo, 135-8550 Japan; 2 Department of Computational Biology and Medical Sciences, Graduate School of Frontier Sciences, The University of Tokyo, Tokyo, 108-8639 Japan; 3 Laboratory of Proteome Research, National Institute of Biomedical Innovation, Health and Nutrition, Osaka, 567-0085 Japan

## Abstract

*ROS1* rearrangement is observed in 1–2% of non-small cell lung cancers (NSCLC). The ROS1 tyrosine kinase inhibitor (TKI) crizotinib has induced marked tumour shrinkage in *ROS1*-rearranged cancers. However, emergence of acquired resistance to TKI is inevitable within a few years. Previous findings indicate that cabozantinib overcomes secondary mutation–mediated crizotinib-resistance in *ROS1*-fusion-positive cells. Here we attempted to establish cabozantinib-resistant cells by *N*-ethyl-*N*-nitrosourea mutagenesis screening using CD74-ROS1–expressing Ba/F3 cells. Two resistant cell lines with CD74-ROS1 F2004V or F2075C mutations, which are homologous to ALK F1174 or F1245 mutations, survived in the presence of a low dose of ROS1-TKI. Removal of ROS1-TKI from these TKI-addicted cells induced excessive activation of ROS1 tyrosine kinase followed by apoptosis. We succeeded in recapturing the TKI-addicted phenotype using doxycycline-inducible CD74-ROS1 mutant over-expression in Ba/F3 cells, suggesting that excessive *ROS1* oncogenic signaling itself induced apoptosis instead of cell growth. Phosphoproteomic analysis and high-throughput inhibitor screening revealed that excessive ROS1 signaling in the TKI-addicted cells phosphorylated or activated apoptosis-related molecules such as FAF1 or p38. Collectively, our findings partly clarify molecular mechanisms of excessive *ROS1* oncogenic signaling that mediates paradoxical induction of apoptosis.

## Introduction

*ROS1* was initially identified as the homolog of the proto-oncogene *v*-*ros*, the transforming gene of the avian sarcoma virus UR2^1^. ROS1 is a receptor tyrosine kinase expressed in the kidney, cerebellum, peripheral neural tissue, stomach, small intestine and colon of adult humans^2^. However, the biological functions of human ROS1 are poorly understood. Chromosomal rearrangements in the kinase domain of ROS1 lead to constitutive activation of ROS1 signaling, followed by phosphorylation of SHP-2 (tyrosine phosphatase, non-receptor type 11) and downstream signaling pathways such as MEK/ERK, JAK/STAT or PI3K/AKT^3, 4^. The aberrant activation of these signaling pathways by *ROS1* fusions contributes to malignant transformation, such as in 1 to 2% of non-small cell lung cancer (NSCLC)^5, 6^. *Anaplastic lymphoma kinase* (*ALK*) which belongs to the same kinase family, shares a high homology with *ROS1* ^7^. Consistent with their high homology, *ROS1*- and *ALK*-rearranged NSCLCs have been treated with shared tyrosine kinase inhibitors (TKIs). Based on *in vitro* or *in vivo* data, a number of ALK-TKIs such as crizotinib (PF-02341066)^8, 9^, ceritinib (LDK378)^10^, lorlatinib (PF-06463922)^11^, or entrectinib (RXDX-101)^12^ have been tested in clinical trials to treat *ROS1*-rearranged NSCLCs. Among these TKIs, crizotinib is approved as first-line therapy for advanced *ROS1* fusion-positive NSCLC by the U.S. Food and Drug Administration and EU European Medicines Agency, based on favourable results in clinical trials^9^. However, emergence of acquired resistance is expected within a few years. To date, acquired resistance to crizotinib has been reported in clinical studies because of the secondary S1986Y/F^13^, G2032R^14^ and D2033N^15^ mutations in *ROS1*.

The acquisition of TKI resistance other than by secondary mutations has been reported in oncogene-driven lung cancers, such as crizotinib resistance mediated by amplification of the *ALK* fusion gene in NSCLC^16^, gefitinib (an epidermal growth factor receptor[EGFR] TKI) resistance mediated by activation of a bypass pathway through *MET* amplification or activation in EGFR-positive NSCLC^17, 18^, or ceritinib resistance mediated by the over-expression of ABCB1 in *ALK*-rearranged NSCLC^19^. However, many characteristics of drug-resistant cancers remain unknown.

To elucidate mechanisms underlying acquired resistance to crizotinib in cancer cells harbouring the *ROS1* fusion gene, we previously performed *N*-ethyl-*N*-nitrosourea (ENU) mutagenesis screening of Ba/F3 cells expressing CD74-ROS1 and identified multiple crizotinib resistant mutations including G2032R solvent front mutation and demonstrated that cabozantinib effectively inhibited the G2032R ROS1 mutation^20^. Based on those results, we attempted to investigate mechanisms underlying acquired resistance to cabozantinib in cancer cells harbouring CD74-ROS1, which is the most common of the *ROS1* fusions^2, 5, 21^. In the process of ENU mutagenesis screening for cabozantinib resistance, we found two CD74-ROS1 mutant clones (F2004V and F2075C) that have a highly activated ROS1 kinase. These clones were intermediately resistant to cabozantinib but, surprisingly, could not survive in the total absence of cabozantinib because of their own excessive ROS1 signaling. They could grow only in the presence of low doses of ROS1-TKIs which controlled their ROS1 kinase activity to an appropriate level. In a sense, they were ‘addicted’ to the presence of ROS1-TKIs.

These findings of, as it were, ‘TKI addiction’ have been reported in several studies^22–26^. TKI-addicted cells commonly have a high activity of oncogene signaling because of gene amplification or point mutations. Furthermore, apoptosis, cell cycle arrest or senescence of these cells seem to be induced by their excessive oncogene signaling. Taken together, our findings and those of others suggest that there is an optimal intensity of oncogene signaling required for survival of cancer cells. Interestingly, similar concepts have been observed in other pathologic states, such as the requirement for an acceptable redox environment defined by oxidative stress levels in striated muscle or the constraint of maintaining methyl-CpG-binding protein 2 (MeCP2) within a certain range of expression. Overexpression of MeCP2 causes MeCP2 duplication syndrome, and loss of function of MeCP2 causes Rett syndrome^27, 28^. As the different example, antiandrogen withdrawal syndrome is observed in some prostate cancer patients. The withdrawal of antiandrogen drugs is prone to decrease serum PSA (prostate specific antigen) and to show the therapeutic effect in some prostate cancer patients^29^.

In the present study, by ENU mutagenesis screening, we identified cells that harbour CD74-ROS1 which were not only resistant to but also addicted to ROS1-TKIs. We also found that ROS1 signaling was excessively activated in these cells by removal of the ROS1-TKI, inducing apoptosis mainly in a caspase-8-dependent manner. We recaptured the TKI-addiction phenotype by conditionally over-expressing the CD74-ROS1 F2075C mutant in Ba/F3 cells harbouring wild-type CD74-ROS1. Our data from a phosphoproteomic analysis identified apoptosis-related molecules which were phosphorylated when ROS1-TKI was removed. Our data from high-throughput inhibitor screening then identified compounds which could keep the ROS1-TKI–addicted cells alive upon removal of the TKI. Our findings may lead to elucidation of some as yet undefined aspects of drug-resistant cancer cells.

## Results

### Establishment of ROS1-TKI–addicted cells by ENU mutagenesis screening

**Figure 1:**
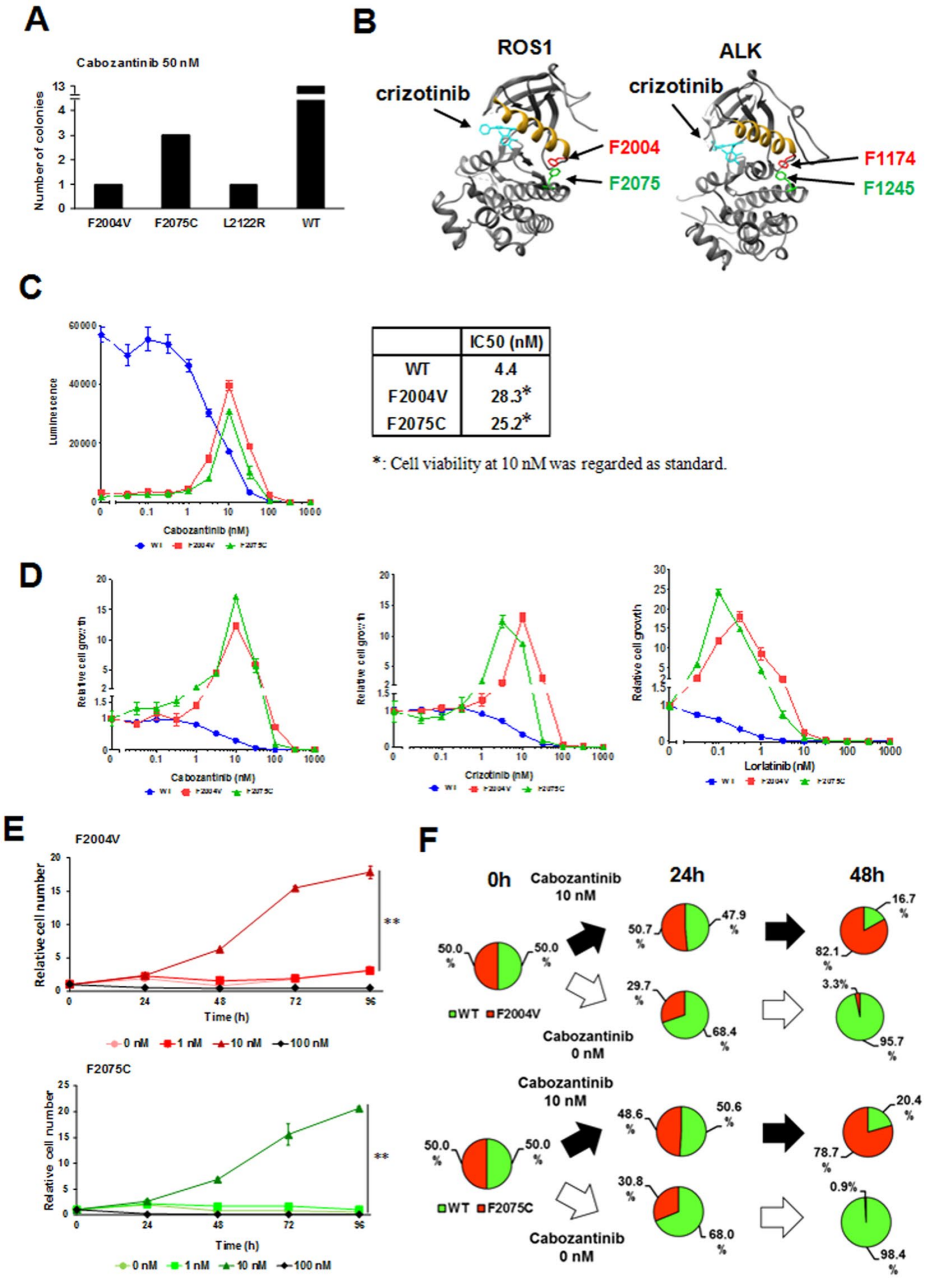
Identification of cabozantinib-resistant mutations by ENU mutagenesis screening and addiction to ROS1-TKIs in Ba/F3 cells expressing F2004V- or F2075C-mutated CD74-ROS1. (**A**) The number of clones harbouring ROS1 kinase domain mutations found by ENU mutagenesis screening. (**B**) Three-dimensional mapping identifying ROS1 mutations based on the crystal structure of ROS1 (left: PDB 3ZBF) or ALK (right: PDB 2XP2) kinase domain in the presence of crizotinib. Mutated residues are shown in red and green in the ROS1 kinase domain and the correspondingly kinase domain. The alpha-C helix motif is depicted in yellow. Figures were drawn using UCSF Chimera software. (**C**) Sensitivity of Ba/F3 cells expressing wild-type (WT), or F2004V- or F2075C-mutated CD74-ROS1 to cabozantinib, shown by luminescence intensity (left). Calculated IC50 values (nM) of each cell line for cabozantinib are shown (right). Each cell line was treated with the indicated dose ranges of inhibitors for 72 h, then cell viability was measured by the CellTiter-Glo assay. (**D**) Sensitivity to cabozantinib (left: relative indication of Fig. 1C), crizotinib (middle) or lorlatinib (right) of Ba/F3 cells expressing wild-type CD74-ROS, or F2004V- or F2075C-mutated CD74-ROS1. Each cell line was treated with the indicated dose ranges of inhibitors for 72 h, then cell viability was measured by the CellTiter-Glo assay. Each value was normalised with the value from untreated cells. (**E**) Cell growth of F2004V- (top) or F2075C- (bottom) mutated cells with each dose of cabozantinib was measured by the CellTiter-Glo assay. Relative cell numbers were indicated by normalizing viability of each cell line with corresponding values at 0 h. Statistical significance was calculated by a *t* test, and ** indicates p < 0.001. (**F**) The dominant populations of wild-type CD74-ROS1, F2004V- (top), or F2075C- (bottom) mutated cells in the presence of 0 or 10 nM cabozantinib for 0, 24 or 48 h, analysed by flow cytometry (dot plots are shown in Supplementary Fig. S2). F2004V- or F2075C-mutated cells were stained with red (PKH26) and wild-type CD74-ROS1 cells with green (PKH67) fluorescent dye.

To explore the cabozantinib-resistant mutations in ROS1 and to find drugs overcoming these mutations, we attempted to establish cabozantinib-resistant Ba/F3 cells harbouring a mutated *CD74*-*ROS1* gene by ENU mutagenesis screening from a single clone of wild-type CD74-ROS1–expressing Ba/F3 cells as previously isolated^20^. After 4 weeks of culture of ENU-treated Ba/F3 cells in the presence of 50 nM cabozantinib, we found 3 distinct mutations (F2004V, F2075C and L2122R) in the ROS1 kinase domain in the isolated clones (Fig. 1A). Among these mutant clones, cells with the F2004V (with a phenylalanine-to-valine substitution at the 2004 residue) or F2075C (with a phenylalanine-to-cysteine substitution at the 2075 residue) mutant CD74-ROS1 could not survive without a low-dose (around 3 to 10 nM) of cabozantinib. These phenylalanine residues in CD74-ROS1 mutants are analogous to phenylalanine at the 1174 or 1245 residue in ALK, located in the alpha-C helix in the N-lobe or the C-lobe, respectively (Fig. 1B). These residues are known to play an important role in stabilizing an inactive conformation of the kinase mediated by hydrophobic interaction^30–32^. We hypothesized that the ROS1-TKI addiction characteristics of these mutant clones were mediated by each corresponding point mutation. Thus, we performed cell viability assays for Ba/F3 cells expressing wild-type, F2004V-, or F2075C-mutated CD74-ROS1. Since the cabozantinib-addicted mutant Ba/F3 cells died when TKI was removed, for F2004V- or F2075C-mutated cells, IC50 values were calculated by comparing the cell viability with 10 nM of cabozantinib; for wild-type CD74-ROS1 cells, the IC50 value was calculated without cabozantinib. F2004V- and F2075C-mutated cells were 6.4- and 5.7-fold resistant to cabozantinib, respectively, compared with wild-type CD74-ROS1 cells. The viability of these mutants was drastically decreased in lower concentrations of cabozantinib (from 0 to 1 nM) (Fig. 1C). Treatment with other ROS1-TKIs such as crizotinib or lorlatinib also showed a similar trend as observed with cabozantinib treatment (Fig. 1D,E and Supplementary Fig. S1A–C). To investigate how the wild-type and TKI-addicted cells behave with or without cabozantinib, we prepared fluorescent-labelled wild-type CD74-ROS1 and different colour fluorescent-labelled F2004V- or F2075C-mutated Ba/F3 cells. Equivalently co-cultured F2004V- or F2075C-mutated cells with wild-type CD74-ROS1 cells were monitored in the presence or absence of 10 nM of cabozantinib. In the presence of 10 nM-cabozantinib, the population of F2004V- or F2075C-mutated cells became dominant at 48 h. By contrast, the population of wild-type CD74-ROS1 cells was dominant in the absence of cabozantinib at 48 h (Fig. 1F and Supplementary Fig. S2).

These results demonstrate that the isolated Ba/F3 cells harbouring F2004V or F2075C CD74-ROS1 mutations were not only resistant but also addicted to ROS1-TKIs, so that they were able to survive only in the presence of a ROS1-TKI.

### Paradoxical apoptosis induction after excessive ROS1 activation by ROS1-TKI removal from TKI-addicted cells

**Figure 2:**
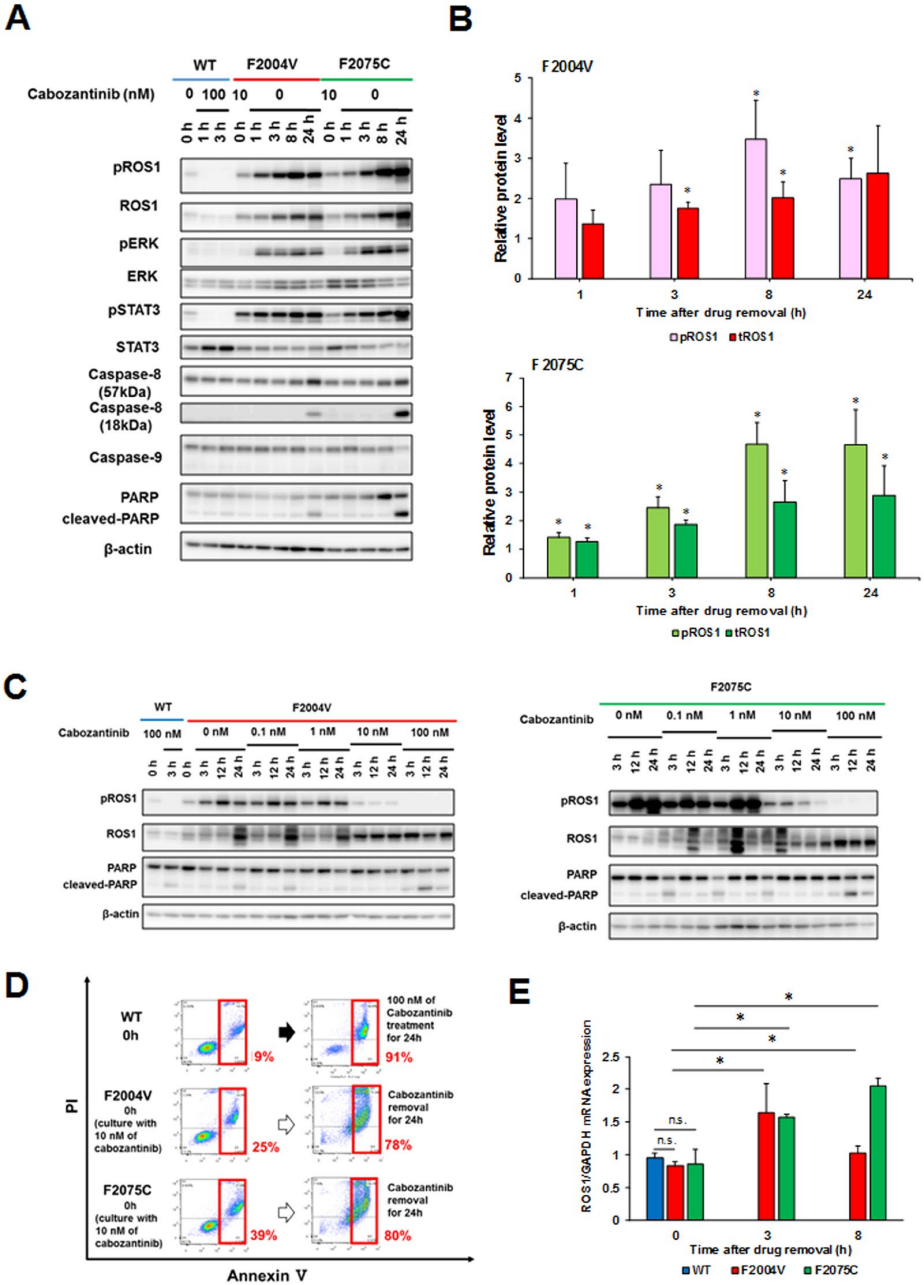
Increased ROS1 activity after removal of ROS1-TKI induces apoptosis. (**A**,**C**) Immunoblotting for F2004V- or F2075C-mutated cells without (**A**) or with cabozantinib at the indicated concentrations (**C**), or wild-type CD74-ROS1 cells treated with 100 nM of cabozantinib. Cell lysates were immunoblotted to detect the indicated proteins. (**B**) The graph indicates protein levels of total or phospho-ROS1 normalised with β-actin. The value at each time point is compared with F2004V-(top) or F2075C- (bottom) mutated cells in the initial state (0 h, in the presence of 10 nM cabozantinib); values are shown as mean ± standard deviation (SD) of three independent experiments (including the values of Fig. 2A). Statistical significance was calculated by a *t* test; * indicates p < 0.05 (compared with values at 0 h). (**D**) Apoptosis assay by flow cytometry for 100 nM cabozantinib-treated wild-type CD74-ROS1 cells and F2004V- or F2075C-mutated cells after removal of cabozantinib for 24 hours. The percentage of cells undergoing apoptosis is shown in red. (**E**) Expression levels of *ROS1* or GAPDH mRNA in wild-type CD74-ROS1, F2004V- or F2075C-mutated cells were quantified using qRT-PCR. The graph indicates relative mRNA expression levels of each *ROS1* that was normalised with *GAPDH*. Each value is shown as mean ± SD of three experiments. Statistical significance was calculated by a *t* test; * indicates p < 0.05 and n.s., p ≥ 0.05. Uncropped blots are presented in Supplementary Fig. S11.

To investigate changes in ROS1 activity and its downstream signaling pathways in F2004V- or F2075C-mutated cells after removal of the ROS1-TKI, we performed immunoblot analysis. When the ROS1-TKI was removed from these cells, expression and phosphorylation of CD74-ROS1 were excessively upregulated, and phosphorylation of ERK and STAT3 were also upregulated in a time-dependent manner (Fig. 2A and B). Consistent with the results of the cell viability assay, PARP, a mediator of apoptosis, was cleaved when the ROS1-TKI was removed from F2004V- or F2075C-mutated cells (Fig. 2A). Interestingly, caspase-8 but not caspase-9 was activated 24 h after removal of cabozantinib (Fig. 2A). In the presence of 10 nM cabozantinib or crizotinib, expression and phosphorylation of ROS1 were normally regulated in F2004V- or F2075C-mutated cells, and PARP was not cleaved. On the other hand, with both higher (100 nM) and lower (0, 0.1 or 1 nM) doses of the TKI, PARP was cleaved within 24 h (Fig. 2C and Supplementary Fig. S3A). We next performed an apoptosis assay to confirm whether these results were consistent with the cleavage of PARP in immunoblot analysis. Apoptotic cell death was observed in F2004V- or F2075C-mutated cells within 24 h after removal of cabozantinib (Fig. 2D). To investigate whether the increased expression of ROS1 was mediated at the transcriptional or posttranscriptional level, we performed quantitative real-time reverse transcription polymerase chain reaction (qRT-PCR) using F2004V- or F2075C-mutated cells. The ROS1 mRNA levels in F2004V- or F2075C-mutated cells were upregulated by TKI removal (Fig. 2E). To check the protein stability of the CD74-ROS1 protein after ROS1-TKI removal, the cells were treated with cycloheximide (CHX) to inhibit *de novo* protein synthesis and by immunoblot. F2004V- or F2075C-mutated cells were pretreated with CHX for 3 h prior to removing cabozantinib. Compared with low-dose cabozantinib treatment, after removal of the TKI, the degradation of ROS1 proteins in both F2004V- and F2075C-mutated cells was delayed (Supplementary Fig. S3B). These results suggested that increased expression of ROS1 was mediated at both the transcriptional and posttranscriptional levels. Furthermore, ROS1-TKI removal upregulated phospho-ROS1 and its downstream growth signaling. At the same time, apoptotic pathways were also activated by ROS1 signaling (Fig. 2A).

**Figure 3:**
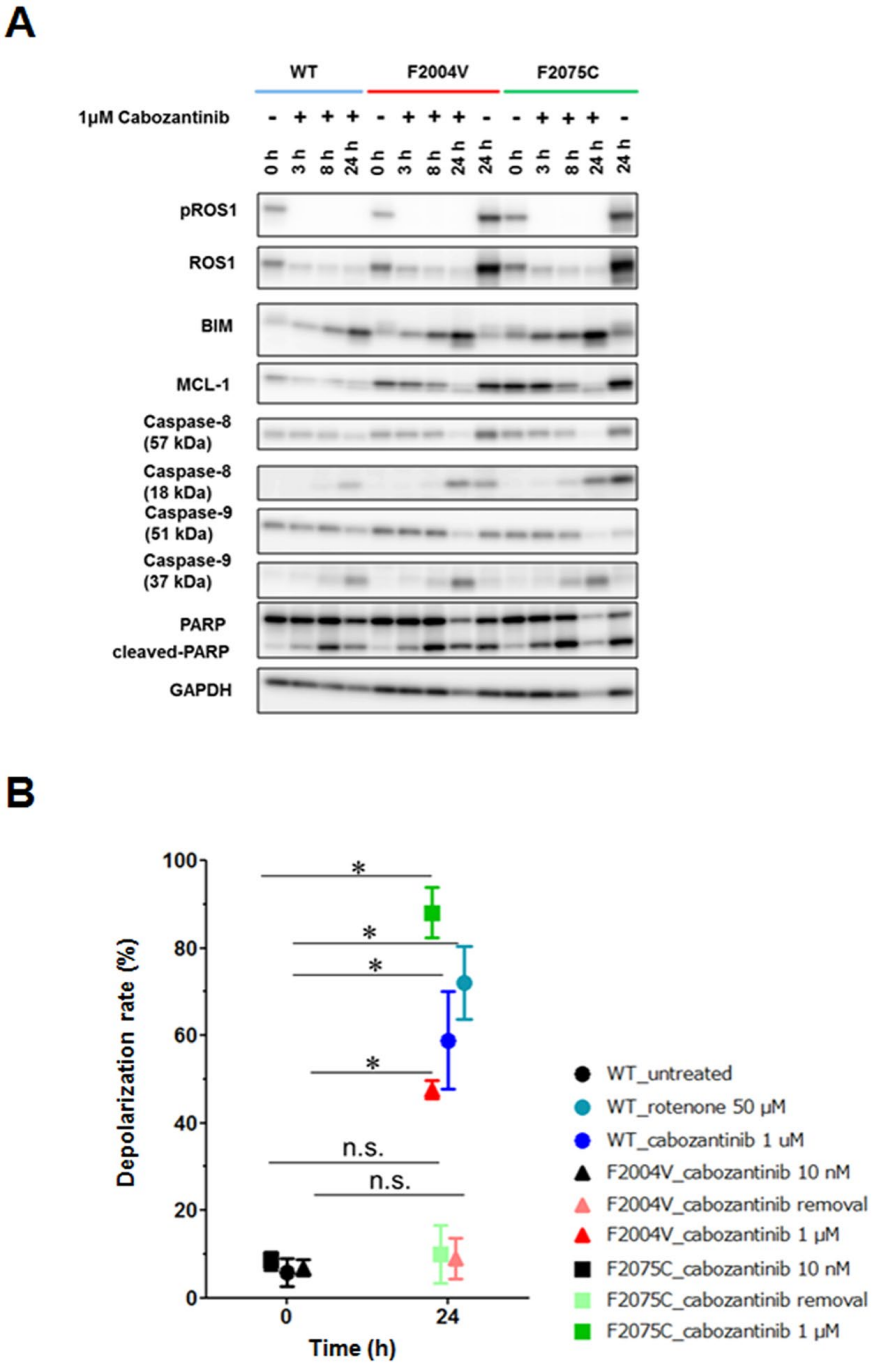
Distinct apoptosis mechanisms with cabozantinib removal and with high-dose cabozantinib treatment. (**A**) Immunoblotting for wild-type CD74-ROS1, F2004V-, or F2075C-mutated cells with or without treatment with 1 μM cabozantinib. Cell lysates were immunoblotted to detect the indicated proteins. (**B**) Mitochondrial depolarization assay by flow cytometry was performed for wild-type CD74-ROS1 cell (treated with 50 μM rotenone or 1 μM cabozantinib), F2004V- or F2075C-mutated cells (with or without 1 μM cabozantib). Rotenone, a mitochondrial electron transport system inhibitor, was used to treat wild-type CD74-ROS1 cells as a positive control. Each value is shown as mean ± SD of three independent experiments. Statistical significance was calculated by a *t* test; * indicates p < 0.05 and n.s., p ≥ 0.05. Uncropped blots are presented in Supplementary Fig. S11.

It has been reported that inhibition of ERK activity in cancer cells harbouring a driver oncogene induces upregulation of BIM (BCL2 like 11)^33–35^. The BH3 domain containing proteins such as BIM is known to activate BAX or BAK, mediators of cytochrome *c* efflux from mitochondria by forming complexes^36^. Cytochrome *c*, an apoptosis-inducing factor, forms complexes with caspase-9 and apoptotic protease-activating factor 1 to form the apoptosome, which then activates caspase-3 to initiate apoptosis^37^. In F2004V- or F2075C-mutated cells, high doses (1 μM) of cabozantinib induce apoptosis mediated by caspase-9 cleavage along with upregulation of BIM and downregulation of MCL-1 (the apoptosis regulator and counterpart of BIM)^38^, whereas removal of cabozantinib induced apoptosis not accompanied by caspase-9 cleavage or changes in BIM or MCL-1 expression (Fig. 3A). When apoptosis is initiated in mitochondria, the mitochondrial membrane is depolarised^39^. Therefore, we next performed a mitochondrial depolarization assay using flow cytometry. Wild-type CD74-ROS1, F2004V- and F2075C-mutated cells were treated with or without 1 μM of cabozantinib or with 50 μM of rotenone, a mitochondrial electron transport system inhibitor as a positive control. The cells were stained with JC-1, the mitochondrial membrane potential probe. This assay revealed that the mitochondrial membranes in both F2004V- and F2075C-mutated cells were significantly depolarised by high doses of cabozantinib, whereas cabozantinib removal led to only slight depolarization (Fig. 3B). These results showed that apoptosis in F2004V- or F2075C-mutated cells was executed through distinct mechanisms, depending on the presence of a high-dose of cabozantinib or its absence.

### Ba/F3 cells conditionally over-expressing CD74-ROS1 F2075C mutant recapture the ROS-TKI–addiction phenotype

**Figure 4:**
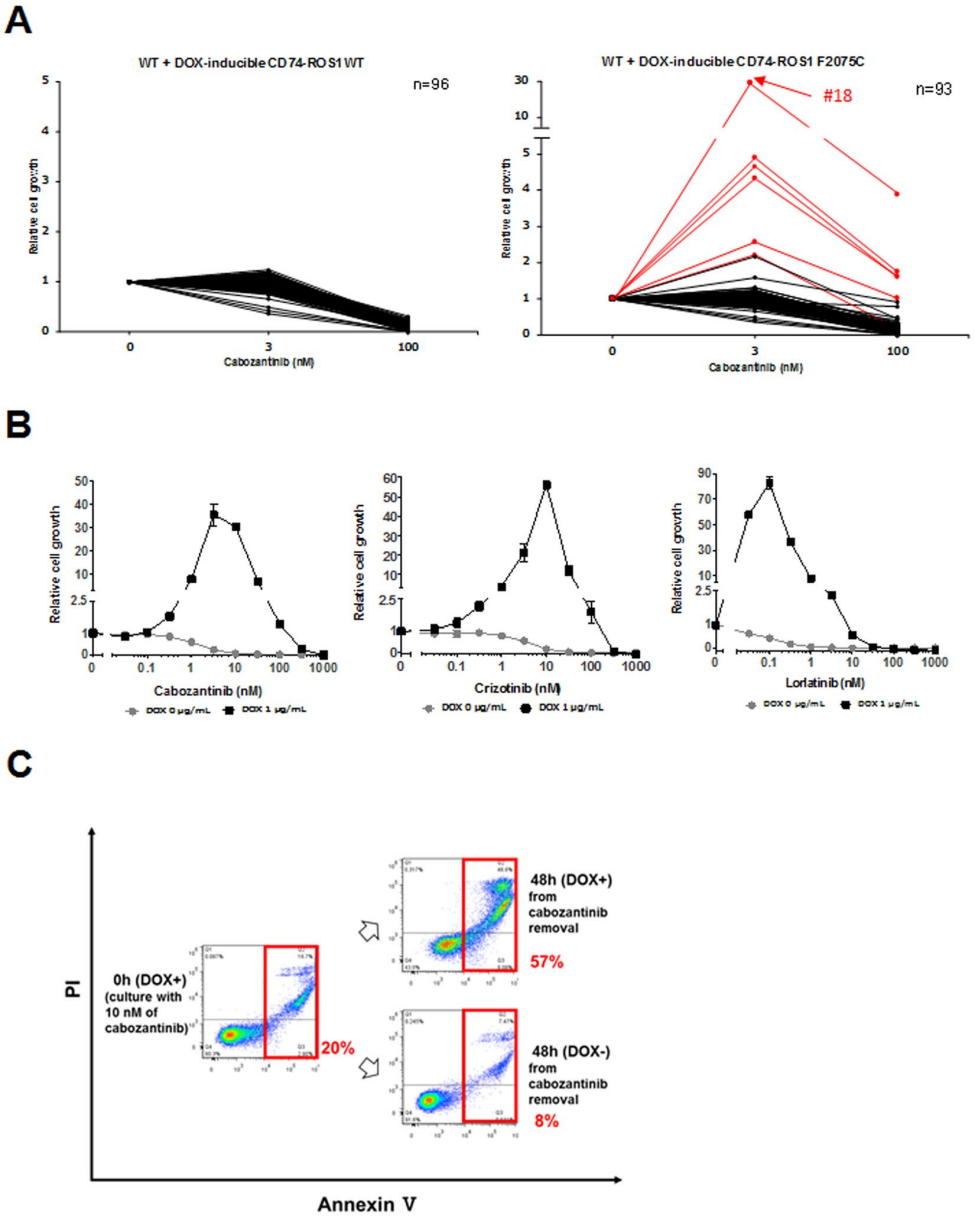
Reconstructed Ba/F3 cells conditionally over-expressing F2075C-mutated CD74-ROS1 show characteristics of ROS1-TKI addiction. (**A**) Viability of wild-type CD74-ROS1 cell clones expressing doxycycline (DOX)-inducible CD74-ROS1 wild-type (left) or F2075C (right). Each clone was untreated (0) or treated with 3 or 10 nM cabozantinib in addition to 1 μg/mL of DOX for 72 hours, and cell viability was measured with the CellTiter-Glo assay. Each value is shown as relative cell growth by normalizing with the value of untreated wells. The top 6 clones with enhanced proliferation under treatment with 3 nM of cabozantinib compared with untreated cells are shown in red. (**B**) Sensitivity to cabozantinib (left), crizotinib (middle) or lorlatinib (right) of wild-type CD74-ROS1 cells expressing DOX-inducible CD74-ROS1 F2075C, clone #18 (reconstructed F2075C). Each reconstructed F2075C cell line with or without 1 μg/mL of DOX was treated with the indicated dose range of inhibitors for 72 h, then cell viability was measured by the CellTiter-Glo assay. Each value is shown as relative cell growth by normalizing to the value of untreated wells. (**C**) Apoptosis assay by flow cytometry for reconstructed F2075C cells incubated with 10 nM cabozantinib and 1 μg/mL DOX for 7 d, then removal of cabozantinib in the presence or absence of 1 μg/mL DOX for 48 h. The percentage of cells undergoing apoptosis is shown in red.

Our results suggest that apoptosis of the ROS1-TKI–addicted cells was induced by excessive ROS1 activity, and their viability was sustained at a certain level of ROS1 activity by a low-dose of various ROS1-TKIs. To check whether the ROS1-TKI–addiction phenotype was independent of mutations of other genes affected by ENU or if it was dependent on excessive ROS1 signaling by a point mutation in the ROS1 kinase domain, we attempted to establish ROS1-TKI-addicted Ba/F3 cells harbouring CD74-ROS1 using the doxycycline (DOX) inducible conditional over-expression system of the wild-type or F2075C mutant of CD74-ROS1. We chose the pSLIK lentiviral vector, a conditional expression system tightly regulated by DOX^40^. We introduced the pSLIK vector expressing wild-type CD74-ROS1 or F2075C mutants into wild-type CD74-ROS1 Ba/F3 cells (that is, a single clone of Ba/F3 cells expressing wild-type CD74-ROS1) to increase ROS1 signaling above basal levels. Monoclonal cells were obtained by limiting dilution. The result was that the ROS1-TKI–addicted clones were not isolated from DOX-inducible wild-type *CD74*-*ROS*–transduced Ba/F3 cells (Fig. 4A, left). Interestingly, however, limiting dilution of CD74-ROS1 F2075C mutant-transduced Ba/F3 cells did isolate some clones with the ROS1-TKI addiction phenotype in the presence of DOX (Fig. 4A, right). Among these clones, we selected clone #18 as a ROS1-TKI–addicted cell (that is, a reconstructed F2075C-mutated cell) and used it for subsequent experiments. Similar to F2004V- or F2075C-mutated cells established by ENU mutagenesis screening, the viability of reconstructed F2075C-mutated cells was rescued by treatment with a low dose of ROS1-TKIs when CD74-ROS1–F2075C mutant protein expression was induced by DOX treatment (Fig. 4B). Consistent with this result, growth of the reconstructed F2075C-mutated cells in the presence of 10 nM cabozantinib and 1 μg/mL DOX was significantly enhanced compared with culturing with DOX in the absence of cabozantinib (Supplementary Fig. S4A). Apoptosis assay by flow cytometry showed that, in reconstructed F2075C-mutated cells treated with DOX, apoptosis was induced by ROS1-TKI removal. This induction of apoptosis was cancelled by removal of DOX from the reconstructed F2075C-mutated cells (Fig. 4C). These results suggest that a point mutation which potentially activates ROS1 can induce the ROS1-TKI–addiction phenotype, as well as that excessive ROS1 signaling by CD74-ROS1 F2075C over-expression is sufficient to activate apoptotic signaling.

### Phosphoproteomic analysis identified phosphorylation of apoptosis inducing-factors upon excessive activation of ROS1 signaling

The excessive activation of ROS1 in ROS1-TKI–addicted cells might phosphorylate a greater variety of proteins than the normal ROS1 activation level in cells with wild-type CD74-ROS1, potentially leading to aberrant activation of various molecular pathways, including apoptotic signaling. Thus, we performed phosphoproteomic analysis of F2004V- and F2075C-mutated cells.

In the phosphoproteomic analysis, cabozantinib-treated wild-type CD74-ROS1 cells were treated with or without 100 nM of cabozantinib for 1 h, while F2004V- or F2075C-mutated cells (initially cultured with 10 nM of cabozantinib) were treated in the absence or presence of cabozantinib for 1 or 8 h. To compare phosphopeptides derived from excessive activation of ROS1 signaling by cabozantinib removal with those derived from inhibition of ROS1 signaling by cabozantinib, phosphoproteomic data of wild-type CD74-ROS1 cells were used as log2 fold-change values (untreated/100 nM cabozantinib), that is, the detected intensity of phosphopeptides in untreated samples divided by the detected intensity of phosphopeptides in cabozantinib-treated samples. Phosphoproteomic data from F2004V- or F2075C-mutated cells were used as log2-change values (cabozantinib removal/10 nM cabozantinib), that is, the detected intensity of phosphopeptides in samples without cabozantinib divided by the detected intensity of phosphopeptides in samples treated with 10 nM of cabozantinib. This analysis identified approximately 4000 phosphorylation sites in each sample. The detection rates of phosphorylated-serine, threonine, or tyrosine (pY) in these samples were almost equal (Supplementary Table S1A). However, the amount of pY peptides in both F2004V- and F2075C-mutated cells maintained without cabozantinib for 8 h was significantly upregulated (more than mean + 2 SD) from 2.7 in the presence of low-dose cabozantinib to 55.6 (F2004V) and 49.7 (F2075C) in the absence of the TKI, suggesting that large amounts of tyrosine-phosphorylated proteins were produced by excessive ROS1 signaling in F2004V- or F2075C-mutated cells maintained without cabozantinib (Supplementary Table S1B and Figure S5).

**Figure 5:**
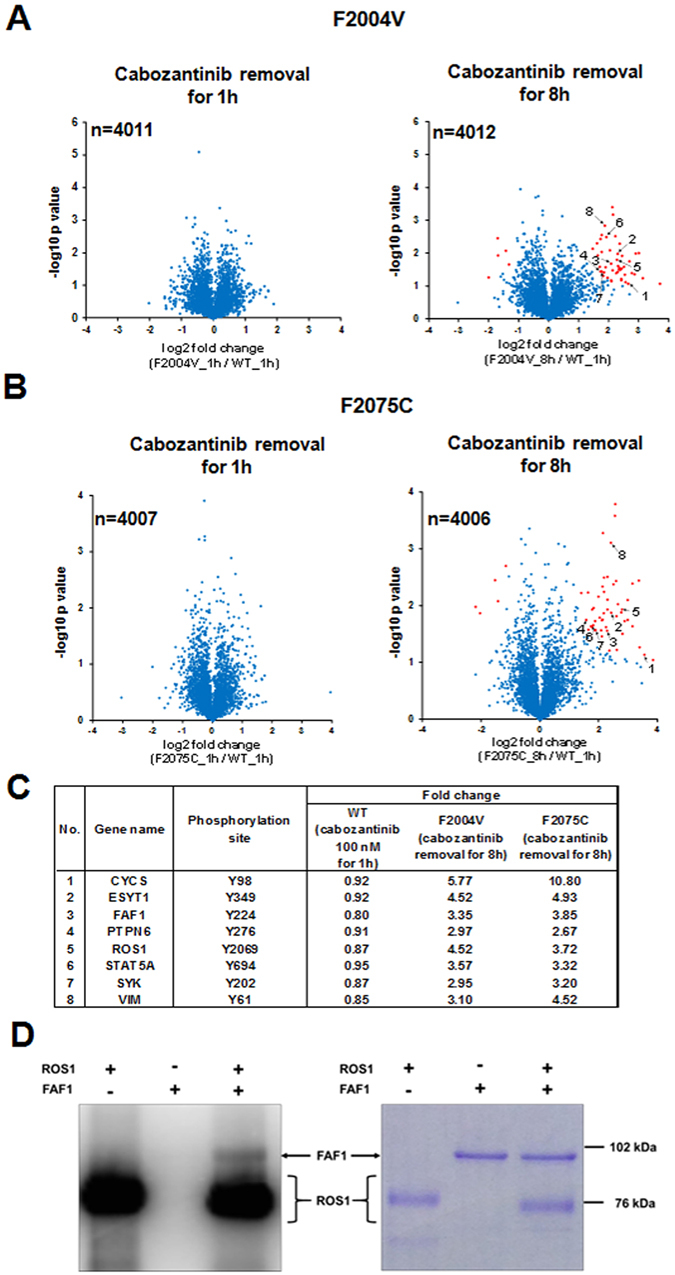
Identification of phosphoproteins phosphorylated by excessive ROS1 signaling. (**A**,**B**) Volcano plot indicates log2-fold-change in values (horizontal axis) of phosphopeptides in F2004V- or F2075C-mutated cells without cabozantinib and cabozantinib-treated (100 nM) wild-type CD74-ROS1 cells, against the −log10 *p* value (vertical axis). These plots indicate the log2 fold-change between upregulated or downregulated phosphopeptides in F2004V- (**A**) or F2075C- (**B**) mutated cells by cabozantinib removal (F2004V or F2075C cells without/with 10 nM cabozantinib, respectively) and upregulated or downregulated phosphopeptides in wild-type CD74-ROS1 cell (untreated/cabozantinib-treated). Each plot is based on the mean fold-change of value and the *p* value of two independent experiments. Plots which passed the 0.05-Benjamini-Hochberg-false discovery rate threshold are shown in red. (**C**) List of representative common phosphopeptides which were significantly upregulated in the F2004V- or F2075C-mutated cells after removal of cabozantinib. (**D**) Coomassie brilliant blue-stained gel (right) and autoradiography (left) of human ROS1 and FAF1 proteins electrophoresed with [gamma-^32^P] ATP. Uncropped gel and autoradiograph are presented in Supplementary Fig. S11.

To identify significantly upregulated or downregulated phosphopeptides, the data obtained by phosphoproteomic analysis were represented in volcano plots based on a two-sample *t* test comparing F2004V (cabozantinib removal/10 nM cabozantinib) or F2075C (cabozantinib removal/10 nM cabozantinib) with wild-type CD74-ROS1 cells (untreated/100 nM cabozantinib) (Fig. 5A and B). In these plots, the Benjamini-Hochberg false discovery rate (BH-FDR) was chosen to evaluate statistical significance. A total of 52 statistically significant phosphopeptides were detected in F2004V-mutated cells and 54 in F2075C-mutated cells (red dots in Fig. 5A and B). Common phosphoproteins derived among the statistically significant phosphopeptides in F2004V- or F2075C-mutated cells and wild-type CD74-ROS1 cells included ROS1 derived by autophosphorylation; ESYT1 (extended synaptotagmin 1)^41^ and PTPN6 (protein-tyrosine phosphatase SHP-1)^42^, known to be ROS1substrates; as well as downstream growth effectors such as STAT5A (signal transducer and activator of transcription A) or SYK (spleen associated tyrosine kinase). Apoptotic factors such as CYCS (cytochrome *c*) or FAF1 (Fas associated factor 1) were also detected. Other than these phosphoproteins, VIM (vimentin) was also detected consistent with reports that the Tyr 61 residue of human or murine VIM is phosphorylated by tyrosine kinases (http://www.phosphosite.org) (Fig. 5A–C).

To check whether FAF1, one of the apoptosis-related factors found in the analysis (Fig. 5C), was phosphorylated by ROS1, we performed an immunoprecipitation assay using anti-FAF1 antibody in F2004V- or F2075C-mutated cells and an *in vitro* kinase assay with recombinant human ROS1 and human FAF1 proteins with radiolabeled-^32^P-ATP. This assay revealed that FAF1 interacted with ROS1 and was tyrosine-phosphorylated when ROS1 signaling was excessively activated by removal of cabozantinib from F2004V-or F2075C-mutated cells (Supplementary Fig. S6A and B). Furthermore, the *in vitro* kinase assay revealed that ROS1 directly phosphorylated FAF1 (Fig. 5D).

These phosphoproteomic data revealed that excessive ROS1 signaling activated a large number of proteins involved in proliferation or apoptosis. Furthermore, consistent with results of the phosphoproteomic analysis, the immunoprecipitation assay or *in vitro* kinase assay showed that FAF1 was tyrosine-phosphorylated by ROS1.

### High-throughput inhibitor screening identified compounds which rescue the viability of ROS1-TKI addicted cells

**Figure 6:**
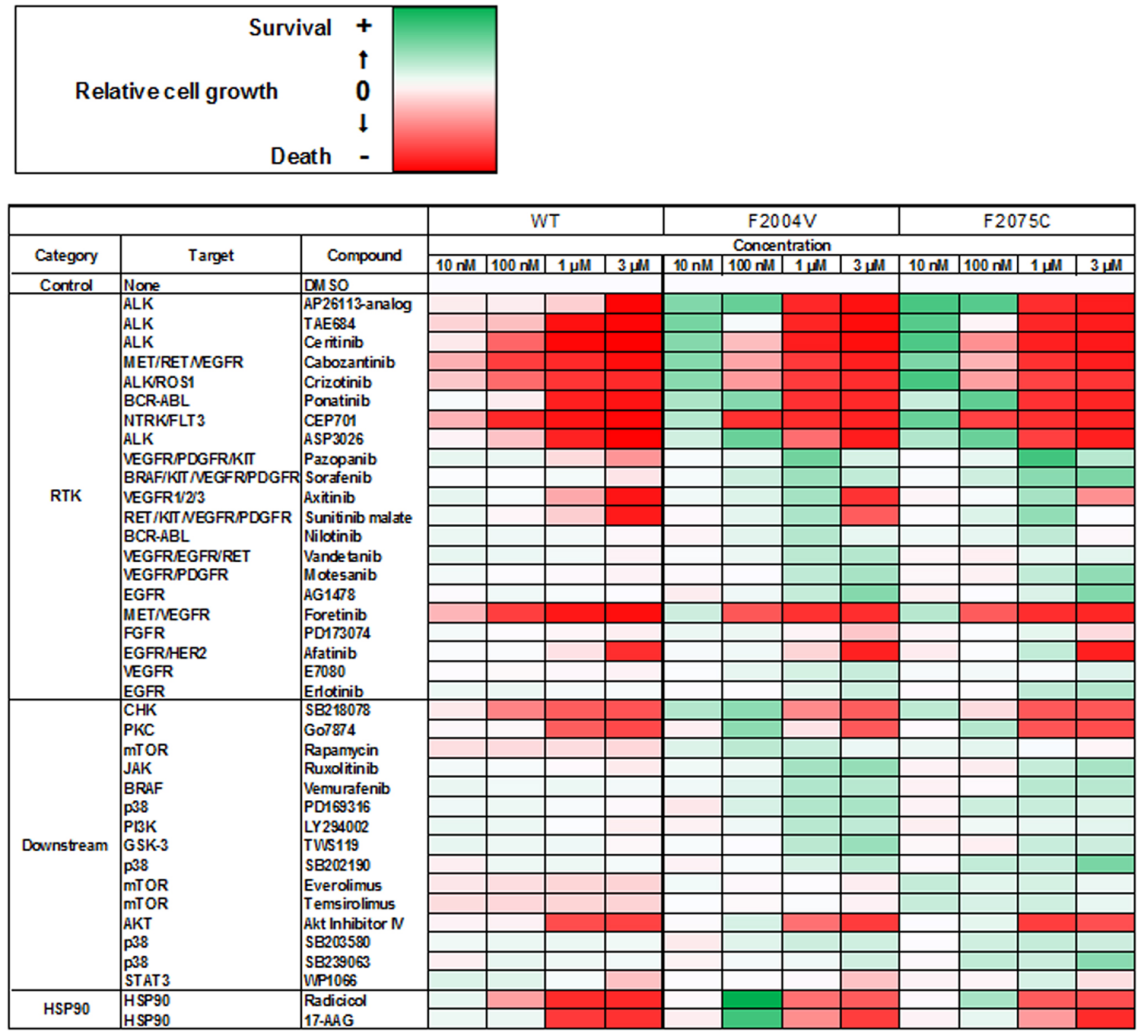
High-throughput inhibitor screening for Ba/F3 cells expressing wild-type CD74-ROS1, F2004V-, or F2075C-mutated CD74-ROS1. Representative data showing cell growth of wild-type CD74-ROS1 (WT), F2004V- or F2075C-mutated cells with treatment by each listed compound with DMSO treatment as control are shown in a heat map (all screening data and detailed values are shown in Supplementary Fig. S7). Red indicates that relative cell growth is suppressed and green that relative cell growth is enhanced compared with DMSO treatment. Cells were treated with 10, 100 nM, 1 or 3 μM of the indicated compounds for 72 h, then cell viability was measured by the CellTiter-Glo assay.

**Figure 7:**
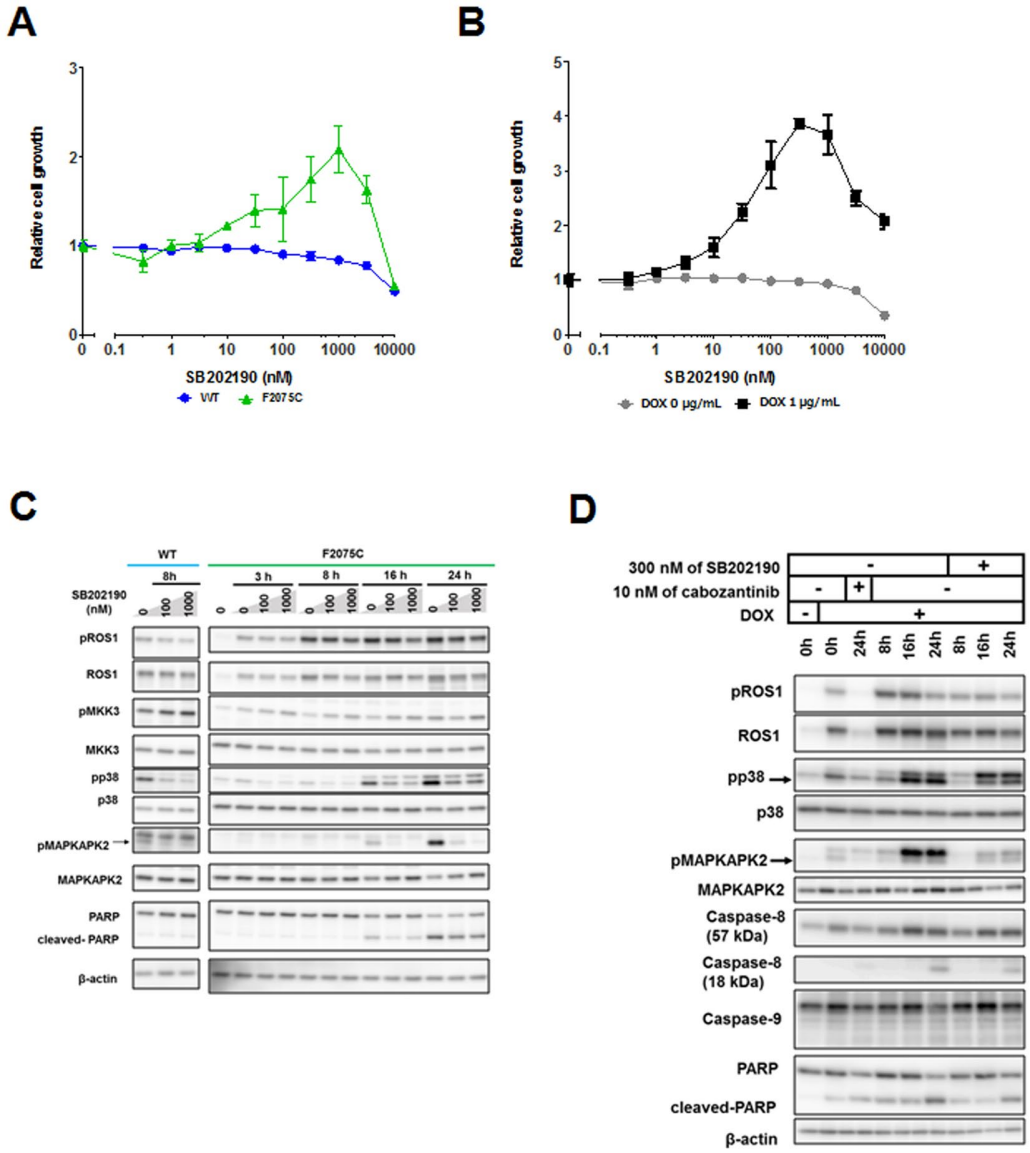
The rescue effect of p38 inhibitor (SB202190) on F2075C-mutated cells obtained by ENU mutagenesis screening or reconstructed F2075C-mutated cells. (**A**,**B**) Effect of SB202190 on wild-type CD74-ROS1, ENU-screened F2075C-mutated cells (**A**), or reconstructed F2075C cells with or without 1 μg/mL DOX (**B**). Each cell line was treated with the indicated dose ranges of inhibitors for 72 h, then cell viability was measured by the CellTiter-Glo assay. Each value is shown as relative cell growth by normalizing with the values of untreated wells. (**C**,**D**) Immunoblotting of SB202190-treated wild-type CD74-ROS1 cells or F2075C-mutated cell (**C**), or SB202190- or cabozantinib-treated reconstructed F2075C-mutated cells with or without 1 μg/mL DOX ﻿﻿(the cells shown DOX (+) were pretreated with DOX for 24 h.)﻿ ﻿(**D**). Cell lysates were immunoblotted to detect the indicated proteins. Uncropped blots are presented in Supplementary Fig. S11.

To identify specific factors related to the induction of apoptosis by excessive ROS1 signaling, we performed high-throughput inhibitor screening. This screening revealed that ROS1-TKIs rescued the viability of F2004V- or F2075C-mutated cells (Fig. 6 and Supplementary Fig. S7). We also found that brigatinib and AP26113-analog (both ALK-TKIs) or ponatinib (AP24534: a multi-targeted TKI) also rescued the viability of F2004V- or F2075C-mutated cells, suggesting that these compounds inhibit ROS1 activity (Fig. 6, Supplementary Fig. S1D–G and S7). Other than these TKIs, mitogen-activated protein kinase 14 (MAPK14: p38) inhibitors such as SB202190 or SB239063 partially rescued the viability of ENU-screened F2075C- or reconstructed F2075C-mutated cells (Figs 6 and 7A,B, Supplementary Figs S4B, S7 and S8A). Consistent with these results, immunoblotting of F2075C-mutated cells obtained either by ENU mutagenesis screening or by reconstruction revealed that p38MAPK signaling in these cells was upregulated by cabozantinib removal, and PARP cleavage was partially suppressed by inhibition of p38 activity by an inhibitor (Fig. 7C,D and Supplementary Fig. S8B). Furthermore, Hsp90 inhibitors such as radicicol (F2004V and F2075C; 30 nM) or 17-AAG (F2004C; 30 nM, F2075C; 300 nM) also partially rescued viability of ENU-screened F2004V-, F2075C- or reconstructed-F2075C mutated cells (Fig. 6, Supplementary Figs S4C, S9A and B). These Hsp90 inhibitors suppressed ROS1 phosphorylation in wild-type CD74-ROS1, F2004V-, or F2075C-mutated cells (Supplementary Fig. S9C–E) similarly to what has been observed in *ROS1*-rearranged lung cancer cells^43^.

Several previous studies suggested that hyperactivation of the MEK/ERK signaling pathway leads to cell cycle arrest, senescence, or apoptosis in cells which have excessively activated driver oncogene signaling^22, 24–26^. We also confirmed that MEK/ERK signaling in F2004V- or F2075C-mutated cells was hyperactivated by excessive ROS1 signaling (Fig. 2A). Thus, we hypothesized that hyperactivated MEK/ERK signaling in F2004V- or F2075C-mutated cells contributes to apoptosis. However, treatment by a MEK inhibitor in the absences of cabozantinib could not rescue F2004V- and F2075C-mutated cells from undergoing apoptosis; even combined treatment with a MEK inhibitor and PI3K inhibitor also could not rescue these cells from apoptosis (Supplementary Fig. S10A and B).

These results suggest that the survival and growth of the ROS1-TKI–addicted cells were potently dependent on the intensity of ROS1 signaling activity and that cell death of ENU-screened F2075C- or reconstructed F2075C-mutated cells was partially dependent, in addition to excessive ROS1 signaling activity, on p38MAPK activity.

## Discussion

Overcoming resistance to cancer therapies is a significant clinical challenge. Intratumour heterogeneity is observed in various subtypes of cancer and is thought to be one of the causes of therapeutic resistance. The mechanisms that underlie emergence of drug-resistant clones are still controversial. Even among NSCLC cell lines harbouring the same driver (EGFR), two different processes have been observed: the acquisition of the drug resistant phenotype by selection of a resistant clone that existed prior to therapy^44^ and the evolution via *de novo* changes during therapy^45^. In the present study, we found certain TKI-resistant cells with unique characteristics, namely that survival of these cells was dependent not on withdrawal of a TKI but rather on continued TKI exposure at low doses. If such a TKI-addicted clone were present among heterogeneous clones in a tumour during molecular targeted therapy, it might emerge as the predominant drug-resistant clone, resulting in a serious threat to the patient. Therefore, elucidating the characteristics of TKI addiction will give us significant clinical findings to overcome the resistance of cancer therapies.

In the present study, we established two lines of ROS1-TKI–addicted cancer cells by ENU mutagenesis screening, namely CD74-ROS1–positive Ba/F3 cells harbouring either F2004V or F2075C mutations. These mutants produce residues in ROS1 that are analogous to phenylalanine at the 1174 or 1245 residue in ALK. *ALK* F1174L/I/S or F1245C mutations have been observed in patients with neuroblastoma^30^. The F1174 residue in the alpha-C helix of the N-lobe and the F1245 residue in the C-lobe in wild-type ALK interact with each other by hydrophobic interaction to regulate the active conformation of ALK.

Therefore, when mutated genes produce either of these residues, the hydrophobic interaction between these sites is disrupted, easily allowing the ALK kinase domain to take on the active conformation^30–32^. Based on these previous studies and our findings in the present study, it is suggested that CD74-ROS1 F2004V or F2075C mutants might influence active conformation of ROS1, leading to higher kinase activity. Therefore, it seems that the ROS1-TKI–addicted cells in the present study had excessive ROS1 activity when the ROS1-TKI was removed. We demonstrated that the viability of these ROS1 TKI-addicted cells was sustained in the presence of low doses of the ROS1-TKIs such as cabozantinib, crizotinib or lorlatinib. These findings suggest that survival and growth of TKI-addicted cells is strongly dependent on the magnitude of ROS1 activity. In the experimental situation that mimicked intratumour heterogeneity with the coexistence of TKI-addicted and non -addicted cells, the population of TKI-addicted cells predominated in the presence of cabozantinib, while that of non-addicted cells predominated in its absence. These observations imply that growth of such TKI-addicted cells is promoted by the presence of TKI, which might therefore induce clinical resistance to molecular targeted therapies. Such TKI-addicted tumour cell clones have not been reported in clinical studies thus far. If they do exist clinically, they may be in numbers too small to be detected in specimens, or it may be difficult to establish those cell lines in the lab because of their unique phenotype, requiring the presence of a TKI to survive and grow.

We next demonstrated that ROS1 phosphorylation and expression were excessively elevated in the ROS1-TKI–addicted cells when the TKI was removed. We observed that both growth and apoptotic signaling were simultaneously activated by excessive ROS1 activation, so that, eventually, the TKI–addicted cells died. The upregulation of ROS1 expression was probably due to reactivation of mTOR (mechanistic target of rapamycin) or PI3K/Akt signaling pathways (pathways related to protein synthesis) when the ROS1-TKI was removed. Our data suggest that aberrant oncogenic ROS1 signaling may activate various signaling pathways, not only those growth signaling but also that which induces apoptosis. The accumulation of aberrant, mutated ROS1 fusion proteins probably contributes to this induction of apoptosis. Therefore, the viability of ROS1-TKI–addicted cells can be sustained by controlling ROS1 signaling in the presence of low doses of ROS1-TKI.

Our data demonstrated by a molecular biological approach that apoptosis of ROS1-TKI–addicted cells by excessive ROS1 signaling seemed to be strongly dependent upon caspase-8 and molecules related to death receptor-dependent apoptosis rather than on caspase-9 and molecules related to apoptosis originating from the mitochondria, suggesting that the apoptotic mechanism induced by excessive ROS1 signaling differs from that caused by high concentrations of ROS1-TKIs during cancer treatment. These unique mechanisms mediated by excessive ROS1 signaling might have novel therapeutic implications.

We succeeded in establishing the ROS1-TKI–addicted cell line by transferring F2075C-mutated CD74-ROS1 into Ba/F3 cells harbouring wild-type CD74-ROS1. These cells had characteristics similar to the ROS1-TKI–addicted cells we selected by ENU mutagenesis screening. A previous study demonstrated that the accumulation of *NPM*-*ALK* in the cytoplasm induced cell death by excessive ALK signaling in anaplastic large cell lymphoma (ALCL) cells^26^. Although *NPM*-*ALK*-positive cells in that study acquired excessive ALK activity by gene amplification, CD74-ROS1- positive cells in the present study acquired excessive ROS1 activity not by gene amplification but by a point mutation which potentially activates ROS1.

The data from our phosphoproteomic analysis revealed molecules that were phosphorylated by excessive ROS1 signaling, and we estimated that phosphorylated FAF1 contributes to induction of apoptosis. FAF1 is a member of the death-inducing signaling complex, the initiator of Fas- and caspase-8–induced apoptosis^46, 47^. It is reported that human FAF1 is phosphorylated at Tyr225, Ser289, or Ser291 residues (murine FAF1: Tyr224, Ser288 or Ser290) (http://www.phosphosite.org). FAF1 is phosphorylated at Ser289 and Ser291 residues by casein kinase 2 or aurora-A to regulate apoptosis or proteasome-dependent degradation of aurora-A^48, 49^. Although it is reported that the Tyr225 residue of human FAF1 is phosphorylated by NPM-ALK^50^, the biological functions of Tyr225-phosphorylated FAF1 are still undefined. We speculate that FAF1 phosphorylated by ROS1 might play an important role in the apoptosis mediated by excessive ROS1 signaling in ROS1-TKI–addicted cells. However, further studies are needed to more deeply investigate cofactors involving FAF1 or other death-signaling molecules. The results obtained by this phosphoproteomics approach also suggest that various signaling pathways were induced by excessive ROS1 signaling, including but not limited to apoptosis-related events.

The data from the high-throughput inhibitor screening revealed that p38 was a promising molecular target activated by excessive ROS1 signaling and may therefore contribute to induction of apoptosis in ENU screened F2075C- or reconstructed F2075C-mutated cells. The p38MAPK signaling pathway is activated by inflammatory cytokines and cellular stress (such as UV radiation, DNA damage or oxidative stress); it plays roles in differentiation, apoptosis, or autophagy by controlling transcription factors^51^. We observed high activation of this signaling pathway by excessive ROS1 activation in F2075C- or reconstructed F2075C-mutated cells. Furthermore, we demonstrated that inhibition of this pathway by p38 inhibitors partially suppressed apoptosis in these cells that occurs when a ROS1-TKI is removed. These observations suggest that apoptosis by excessive ROS1 signaling is related to the p38MAPK signaling pathway. More detailed analysis is needed to elucidate the mechanisms by which excessive ROS1 signaling activates the p38MAPK pathway. Other than p38 inhibitors, Hsp90 inhibitors also rescued viability of ROS1-TKI–addicted cells. Hsp90 is a molecular chaperone which regulates protein folding and stability^52^. Inhibition of Hsp90 activity inactivates oncogenic signaling pathways such as EGFR^53^, EML4-ALK^16^ or SLC34A2-ROS1^43^ by disrupting the proteasomal degradation of kinases, ultimately suppressing proliferation of cells harbouring these oncogenes. Our findings suggest that Hsp90 inhibitors partially restore the viability of ROS1-TKI–addicted cells by suppressing the excessive ROS1 activity occurring on removal of the ROS1-TKI. This screening approach is promising for exploring key molecules that may induce apoptosis when ROS1-TKIs are removed. Therefore, further investigation is needed to expand the library of inhibitors or to screen the shRNA library, which may further elucidate the characteristics of TKI addiction.

Consistent with our findings, cell death or senescence by excessive oncogenic signaling has been observed in various drug-resistant cancer cells^22–26^. These cells may acquire highly activated oncogenic signaling by such as gene amplification or point mutations to evade molecular targeted therapies. However, excessive oncogenic signaling in the absence of the corresponding TKI might be enough to cause their own cell death or senescence. It may be that there is an optimum intensity of oncogene signaling required for cancer cell growth. We observed that TKI-addicted cell populations were attenuated in the absence of TKI, while those of TKI-sensitive cells were attenuated in the presence of TKI. Contrary to ‘disease flare’ that withdrawal of EGFR-TKI from patients with the mutated *EGFR*-lung cancer progresses the tumours^54^, the effect of ‘ROS1-TKI addiction’ might regress the tumours in the mutated ROS1 fusion-positive NSCLC when ROS1-TKI is withdrawn from the patients. Although such spontaneous tumour regression after TKI withdrawal have not been reported to date in the clinic, and it might be very difficult to detect and proof the existence, it could be occurred if TKI-addicted tumour were existed even in small population in the patient’s tumour. These observations suggest that intermittent dosing, as it were a ‘drug holiday’, may be an effective approach to prolonging drug efficacy by overcoming both TKI-addicted and TKI-sensitive oncogene-addicted clones. Indeed, some *in vivo* studies have shown great promise for this concept, using intermittent dosing for V600E mutated-*BRAF* positive melanoma^24^ or amplified-*NPM*-*ALK* positive ALCL^25, 26^ in mice. A joint NCI-SWOG randomised clinical trial evaluating the efficacy of intermittent compared with continuous dosing of dabrafenib and trametinib in patients with V600E mutated-*BRAF* positive melanoma is ongoing (ClinicalTrials.gov study NCT02196181). Further elucidation of the correlation between progression and the intensity of oncogene signaling is important, so comprehensive phosphoproteomic analysis focusing on kinases or tyrosine-phosphorylated proteins and subsequent functional analysis of TKI-addicted cancer cells are needed. To evaluate the clinical significance of the unique characteristics of TKI addiction, confirmation of this phenotype in patient-derived and further studies using such cells are needed. Furthermore, when we discuss about the usefulness of such ‘drug holiday’ strategies in molecular targeted therapy on the clinical viewpoint, we should pay attention to pharmacokinetics (PK) of individual patients. If patients with lower PKs against a certain TKI have the TKI-addicted clones in their tumours, a fixed TKI-withdrawal period would not eradicate the TKI-addicted clones and be more prone to develop them on the contrary. Therefore, it might be necessary to make drug holiday protocols for the personalized medicine that takes into account PKs for individual patients.

**Figure 8:**
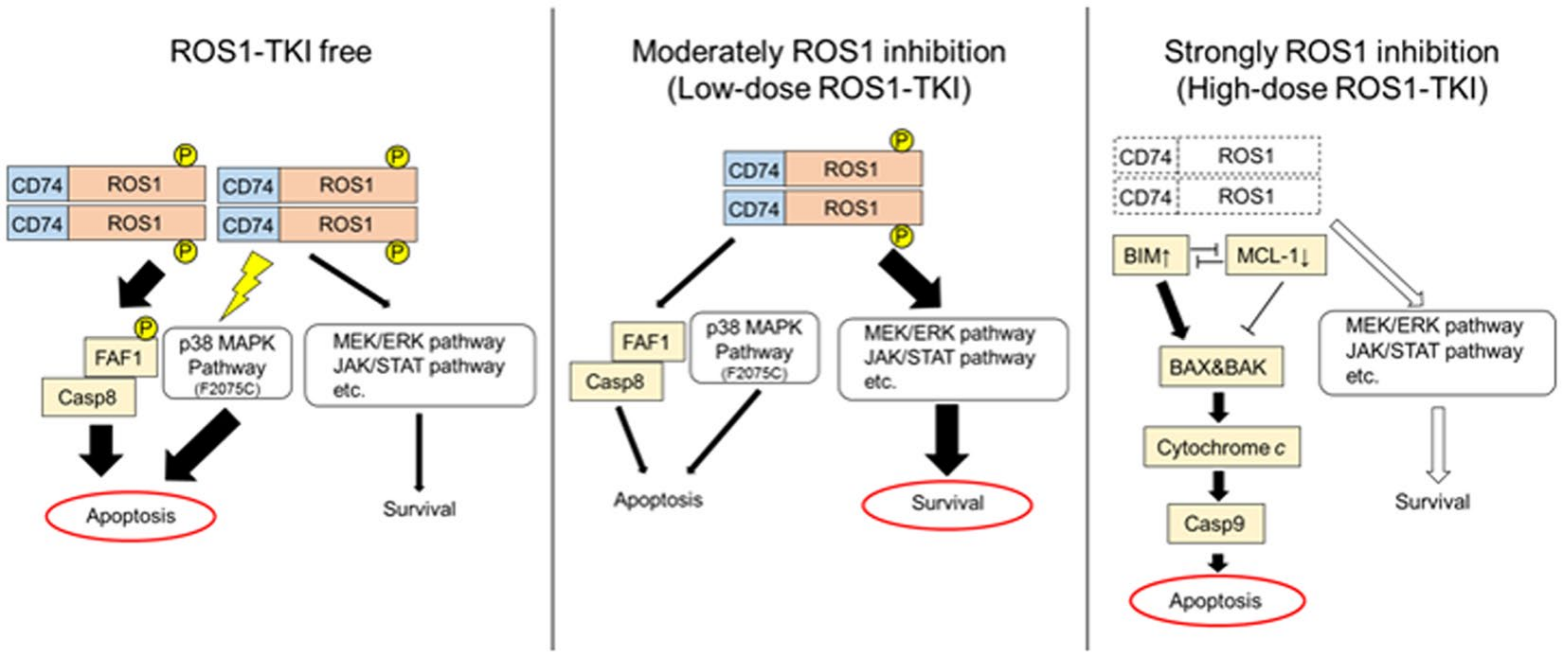
Schematic of cell survival or cell death defined by the intensity of ROS1 activity. (Left) In the absence of a TKI, mutated CD74-ROS1-expressing cells have the potential for excessive activation of ROS1, so that apoptotic signaling is more intense than survival and growth signaling because of excessive ROS1 signaling, that is, when ROS1 activity is not inhibited. (Middle) Cells survive and grow when the intensity of ROS1 activity is moderately inhibited by low-dose ROS1-TKI. (Right) Cells mainly undergo mitochondria originated initiated apoptosis when ROS1 activity is completely inhibited by high-dose ROS1-TKI treatment.

In summary, our study produced several novel findings. We showed that cell survival or death in CD74-ROS1–mutated Ba/F3 cells, which have the potential to excessively upregulate the ROS1 kinase activity, is defined by the balance of ROS1 oncogene signaling intensity (Fig. 8). Furthermore, we succeeded in recapturing TKI addiction phenotype by over-expressing a mutated CD74-ROS1. We also identified candidate molecules which contributed to apoptosis when ROS1 signaling was excessively activated. Our findings provide new insights into the characteristics of drug-resistant cancers and suggest new therapeutic strategies.

## Methods

### Reagents

Crizotinib (PF-02341066) was obtained from ShangHai Biochempartner; cabozantinib (XL184), ceritinib (LDK378), lorlatinib (PF-06463922), trametinib (GSK1120212) from ActiveBiochem; foretinib from AdooQ Bioscience; brigatinib from Sellek; radicicol and rotenone from Cayman Chemical; 17-AAG (17-*N*-Allylamino-17-demethoxygeldanamycin) and GDC-0941 from LC Laboratories; TAE684 from ChemieTek; SB202190 from Cell Signaling Technology; SB239063 from Sigma; and cycloheximide and doxycycline hyclate from Nacalai Tesque. Each compound was prepared in dimethyl sulfoxide (DMSO), ethanol (WAKO) or distilled water for cell culture experiments.

### Cell lines

Ba/F3 cells (RIKEN Bioresource Center), immortalised murine bone marrow-derived pro-B cells, were cultured in Dulbecco’s modified Eagle medium (DMEM, low glucose; WAKO) supplemented with 10% fetal bovine serum (FBS) (D-10) with or without interleukin-3 (IL-3; Invitrogen). Human embryonic kidney 293FT cells (Invitrogen) were cultured in D-10 (high glucose).

When cells transduced with the DOX-inducible lentiviral vectors were cultured, FBS in the medium was replaced with Tet System Approved FBS (Clontech).

Ba/F3 cells expressing F2004V- or F2075C-mutated CD74-ROS1 were cultured with 10 nM of cabozantinib in D-10 based on identification of the appropriate concentration of cabozantinib for cell maintenance by a cell viability assay (Fig. 1C). Wild-type CD74-ROS1 cells were previously isolated as clone #6 from Ba/F3 cells expressing wild-type CD74-ROS1^20^.

Cell lines used in this study were authenticated by short tandem repeat analysis and checked to confirm the absence of mycoplasma contamination at the suppliers.

### Lentiviral infection

For the DOX-inducible expression system, wild-type CD74-ROS1 or F2075C cells were cloned into pEN_TTmcs (Addgene #25755) at the SpeI and XbaI sites and then recombined with pSLIK-Neo (Addgene #25735) using Gateway LR Clonase enzyme (Invitrogen). Recombinant lentiviruses were replicated in 293FT cells by transfecting with packaging plasmids using a lentiviral infection system (ViraPower packaging plasmid: Invitrogen). After retroviral infection into Ba/F3 cells, each cell was selected with G418 (750 μg/mL) for about 10 days.

### ENU mutagenesis screening

Clone #6 from Ba/F3 cells (1.0 × 10^6^ cells/mL) expressing wild-type CD74-ROS1 was treated with 100 μg/mL of ENU (Sigma) for approximately 24 h. All materials that came in contact with ENU were decontaminated with 0.2 M NaOH. After ENU treatment, the cells were washed with phosphate buffered saline (PBS) and incubated for 24 h to allow cell recovery. The ENU-screened cells were placed into five 96-well plates (5 × 10^4^ cells in 200 μL media per well) and selected by cabozantinib at final concentrations of 50, 100, 200, or 500 nM. The cabozantinib-screened cells were cultured up to 4 weeks. The mutated cells were collected and assayed.

### Quantitative real-time reverse transcription polymerase chain reaction (qRT-PCR)

RNA was extracted from cell pellets using RNeasy Mini Kit (QIAGEN). cDNA was synthesized from RNA using the Transcriptor First Strand cDNA Synthesis Kit (Roche). Synthesized cDNA samples were mixed with FastStart Essential DNA Green Master (Roche) and then with the following primers for qRT-PCR: *ROS1*: 5′-TGGAGAAATCAAAGTAGCAGTGA-3′ (forward) and 5′-CATCAGATGTGCCTCCTTCA-3′ (reverse); *GAPDH*: 5′-CGSCTTCAACAGCAACTCCCACTCTTCC-3′ (forward) and 5′-TGGGTGGTCCAGGGTTTCTTACTCCTT-3′ (reverse). qRT-PCR was performed using LightCycler96 (Roche) with the following conditions: 10 min at 95 °C followed by 45 cycles of 15 sec. at 95 °C, 60 sec. at 60 °C, 1 cycle of 10 sec. at 95 °C, 60 sec. at 65 °C, 1 sec. at 97 °C and 1 cycle of 30 sec. at 37 °C. Each sample was measured in triplicate. Quantification was calculated based on a calibration curve obtained from measurement of a standard sample (wild-type CD74-ROS1 cells).

### Cell viability assay

A total of 2000 cells were seeded in 96-well plates in triplicate. After drug treatment for 72 h, the cells were incubated with CellTiter-Glo (Promega) for 10 min at room temperature. Luminescence in wells was measured using a Centro LB 941 microplate luminometer (Berhold Technologies). Data were graphically displayed using GraphPad Prism version 5.0 (GraphPad Software).

Since ENU screened Ba/F3 cells expressing F2004V- or F2075C-mutated CD74-ROS1 died when TKI was removed, the cells were maintained in 10 nM of cabozantinib. After removal of cabozantinib by washing the cells with D-10 (low glucose), the cells were treated with serially diluted cabozantinib for 72 hours.

### Flow cytometry analysis

For the apoptosis assay, cells were stained with Alexa Fluor 488 labelled Annexin-V and propidium iodide with an Alexa Fluor 488 Annexin-V/Dead Cell Apoptosis Kit (Thermo Fischer Scientific) for 15 min at room temperature in the dark. Stained cells were resuspended with the buffer in the kit.

For the mitochondrial depolarization assay, cells were stained with 1 μM of JC-1 dye (Thermo Fischer Scientific) for 30 min at 37 °C in 5% CO_2_. JC-1 stained cells were washed by resuspending with PBS and then. The mitochondrial depolarization rate was defined by green fluorescence in the surviving population as judged from the scatter plot.

For evaluation of the proportions of wild-type CD74-ROS1, F2004V- and F2075C-mutated cells in the presence 0 or 10 nM of cabozantinib, wild-type CD74-ROS1 cells were stained with PKH67 (green fluorescence) and F2004V- and F2075C-mutated cells were stained with PKH26 (red fluorescence, both from Sigma) according to the manufacturer’s instructions. Each population was defined by the corresponding fluorescence intensity in the surviving population as judged from the scatter plot.

Each sample was analysed by flow cytometry with Cytomics FC500 (Beckman Coulter). Output data were using FlowJo software ver. 7.6.5 (FlowJo, LLC).

### Immunoblotting and immunoprecipitation

For immunoblotting, cells were resuspended in lysis buffer containing 100 mM Tris at pH 7.6, 1% SDS, and 10% glycerol and boiled for 5 m at 100 °C. After lysates were cooled on ice, protein concentrations were quantified with the BCA Protein assay kit (Thermo Fischer Scientific) according to the manufacturer’s instructions. Equal amounts of lysate were boiled at 100 °C for 5 min with SDS sample buffer (3% SDS, 65 mM Tris at pH 6.8, 10% glycerol and 0.01% bromophenol blue) and then electrophoresed by sodium dodecyl sulfate-polyacrylamide gel electrophoresis (SDS-PAGE), followed by immunoblotting. We used antibodies against phospho-ROS1 (Tyr2274), ROS1 (D4D6), phospho-p42/44 ERK/MAPK (Tyr202/204), p42/44 ERK/MAPK, phospho-STAT3 (Tyr705), STAT3 (79D7), caspase-8 (D35G2), cleaved-caspase-8 (D5B2), caspase-9 (C9), pAKT (D9E), AKT (C67E7), PARP, BIM, MCL-1 (D35A5), FAF1, p38 MAPK (D13E1), phospho-p38 MAPK (D3F9), MKK3 (D4C3), phospho-MKK3 (D8E9), MAPKAPK2 (D1E11) and phospho-MAPKAPK2 (27B7) (Cell Signaling Technology). Antibodies against GAPDH (6C5) (Millipore) and against β-actin (Sigma) served as loading controls. Quantification of immunoblot results was performed with Multi Gauge ver. 3.0 (Fujifilm). Images were scanned by LAS-3000 (Fujifilm) or Amersham Imager 600 (GE Healthcare) and processed with Photoshop software ver. 7.0 (Adobe) or Multi Gause ver. 3.0 (Fujifilm).

For immunoprecipitation, cells were resuspended in lysis buffer containing 20 mM Tris at pH 7.6, 137 mM NaCl, 1.5 mM MgCl_2_, 0.2% Triton-X, 10% glycerol, 1 mM EDTA, and 1 mM dithiothreitol (DTT), PhosSTOP phosphatase inhibitor cocktail, and Complete mini protease inhibitor cocktail (both from Roche) diluted with distilled water. Lysates were centrifuged at 16,000 × g for 15 min at 4 °C. The supernatant was quantified in the same manner as for immunoblotting. Equal amounts of lysate were incubated with the corresponding antibodies for 1 h at 4 °C. Protein G Mag Sepharose (GE Healthcare) was then added to the mixture, which was incubated overnight at 4 °C on a rotary shaker. After the lysates were bound with antibody-conjugated Protein G Mag Sepharose, they were washed four times using the above lysis buffer, and the bound proteins were eluted with the SDS sample buffer by boiling at 100 °C for 5 min. The SDS samples placed on SDS-PAGE and immunoblotted as described above. Antibodies against ROS1 (69D6; Cell Signaling Technologies) and phosphotyrosine (4G10; Millipore) were used, and mouse IgG was used as a negative control.

### High-throughput inhibitor screening

High-throughput inhibitor screening was performed using the SCADS Inhibitor kit-1, 3 or 4, provided by the Screening Committee of Anticancer Drugs supported by a Grant-in-Aid for Scientific Research on Innovative Areas, Scientific Support Programs for Cancer Research, from the Ministry of Education, Culture, Sports, Science and Technology of Japan. ASP3026 (ChemieTek), TAE684, AEW541 (ActiveBiochem), AP26113-analog (Biochempartner), cabozantinib, foretinib, ponatinib (Selleck), afatinib (BIBW2992, ChemieTek), E7080 (Selleck), motesanib (ChemieTek), CEP701 (Calbiochem) and nintedanib (Selleck) were appended to the library (for a total of 282 compounds). Wild-type CD74-ROS1, F2004V- and F2075C-mutated cells were seeded in triplicate in 96-well plates, and each inhibitor was added at 10, 100 nM, 1, or 3 μM. A DMSO-treated well used in each plate as a control. After 72 h of exposure to the inhibitors, cell viability was measured by the CellTiter-Glo assay.

### *In vitro* kinase assay

Recombinant proteins, human-FAF1 (Abcam) at 0.5 μg and human-ROS1 (Carna Biosciences) at 0.1 μg, were incubated at 30 °C for 1 h with 7.5 μL of kinase buffer containing 20 μM of ATP, 2.5 μCi of [gamma-^32^P] ATP (Perkin Elmer), 250 mM of Tris-HCl (pH 7.6), 50 mM of beta-glycerophosphate, 1 mM of Na_3_VO_4_, 100 mM of MgCl_2_, and Complete mini protease inhibitor cocktail in distilled water. After incubation, the samples were boiled at 100 °C for 5 min with SDS sample buffer and then electrophoresed on SDS-PAGE. The gel was stained by Coomassie brilliant blue, followed by drying and assay for kinase activity by autoradiography. The radiographic images were scanned by Typhoon 9410 (GE Healthcare) and processed with Phostoshop software ver. 7.0.

### Sample preparation for mass spectrometry (MS) analysis

Protein extraction from cell pellets, trypsin digestion and enrichment of phosphopeptides were performed as described previously^55^. Enriched phosphopeptides for each sample were labelled with Tandem Mass Tag (TMT, Thermo Fischer) according to the manufacturer’s protocol at one-tenth scale.

TMT-labelled samples were combined in each set of experiments and then acidified with trifluoroacetic acid. The combined sample was desalted and trapped by octadecyl (C18)-strong cation exchange using StageTip^56, 57^, then fractionated into 7 fractions by elution with each of the buffers as described previously^55^. These 7 fractions of TMT-labelled samples were analysed by liquid chromatography tandem mass spectrometry (LC-MS/MS).

### LC-MS/MS analysis and phosphoproteomic analysis

TMT-labelled fractionated phosphopeptides were analysed by a Q Exactive Plus mass spectrometer (Thermo Fisher Scientific) equipped with an UltiMate 3000 Nano LC system (Thermo Fisher Scientific). Phosphopeptides were separated on a fused silica column (300 mm length × 75 μm inner diameter) packed in-house with the reverse-phase material ReproSil-Pur C18-AQ, 1.9 μm resin (Dr. Maisch, Ammerbuch-Entringen, Germany) at 60*°*C. The mobile phases consisted of buffers A (0.1% formic acid and 2% acetonitrile) and B (0.1% formic acid and 90% acetonitrile). The nanoLC gradient was delivered at 280 nL/min and consisted of a linear gradient of buffer B developed from 5% to 30% B in 85 min.

Raw MS files were processed for detection of peaks, identification and quantification using MaxQuant software ver. 1.5.1.2 supported by the Andromeda search engine^58^. Statistical analysis was performed by Perseus software ver.1.5.0.15. Phosphopeptides were identified from MS/MS spectra by referencing the UniProt mouse database combined with the sequence data of human ROS1 from the UniProt human database. Search results were filtered to a maximum false discovery rate of 0.01 at the protein, peptide and PTM-site levels. We required 2 or more unique or razor peptides for protein identification. PTM sites were considered to be fully localised when they were measured with a localization probability >0.75^59^.

### Statistical analysis

Unless otherwise stated, data are presented as mean ± standard deviation (SD). Statistical analysis was performed using a two-tailed *t* test. Statistical significance was accepted for *p* values < 0.05.

In the two-sample *t* test for phosphoproteomic analysis, a BH-FDR threshold of 0.05 was adopted as the confidence cutoff.

### Data availability

The authors declare that all the data supporting the findings of this study are available within the article or can be provided upon request.

## Electronic supplementary material

Supplementary Information
