# Supplementary material for: TKI-addicted ROS1-rearranged cells are destined to survival or death by the intensity of ROS1 kinase activity
Source: Sci Rep. 2017 Jul 17;7:5519. doi: 10.1038/s41598-017-05736-9 (PMC5514057; doi:10.1038/s41598-017-05736-9)
Supplement: Supplementary file 1 — Supplementary Information [file 41598_2017_5736_MOESM1_ESM.pdf]

Supplementary Information

TKI-addicted ROS1-rearranged cells are destined to survival or death by the intensity of ROS1 kinase activity

Hayato Ogura[1,2], Yuka Nagatake-Kobayashi[1,2], Jun Adachi[3], Takeshi Tomonaga[3], Naoya Fujita[1,2], Ryohei Katayama[1*]

[1] Division of Experimental Chemotherapy, Cancer Chemotherapy Center, Japanese Foundation for Cancer Research, Tokyo 135-8550, JAPAN.
[2] Department of Computational Biology and Medical Sciences, Graduate School of Frontier Sciences, The University of Tokyo, Tokyo 108-8639, JAPAN.
[3] Laboratory of Proteome Research, National Institute of Biomedical Innovation, Health and Nutrition, Osaka 567-0085, JAPAN.

***Corresponding author**
Ryohei Katayama
Cancer Chemotherapy Center, Japanese Foundation for Cancer Research, 3-8-31, Ariake, Koto-ku, Tokyo 135-8550, JAPAN
Phone: +81-3-3520-0111; Fax: +81-3-3570-0484; E-mail: ryohei.katayama@jfcr.or.jp.

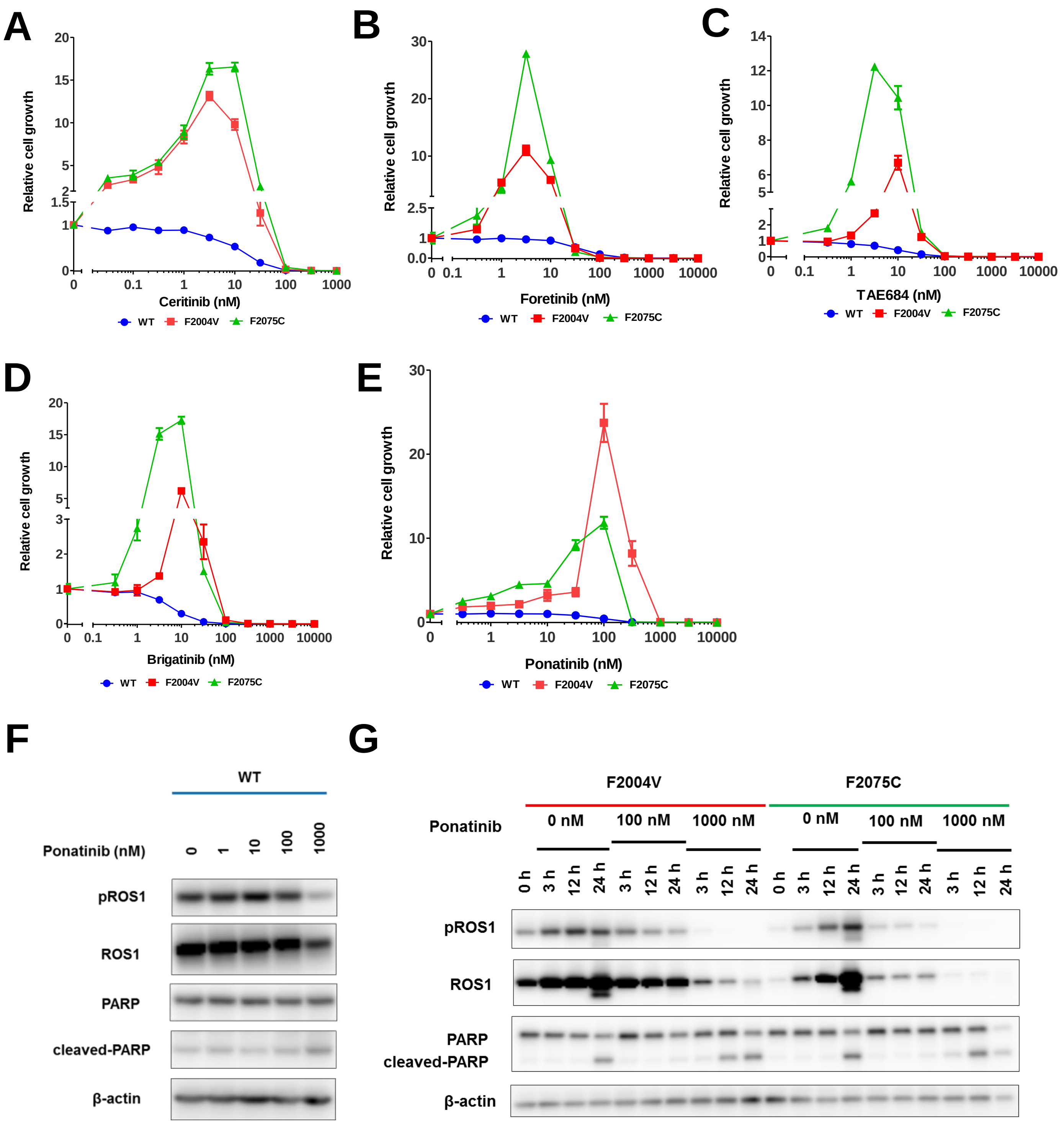

Supplementary figure S1. Sensitivity of wild-type CD74-ROS1, F2004V- or F2075C-mutant cells to ROS1- TKIs or multi-targeted TKIs.

Sensitivity to ceritinib (A), foretinib (B), TAE684 (C), brigatinib (D), or ponatinib (E) of Ba/F3 cells expressing wild-type, F2004V- or F2075C-mutated CD74-ROS1. Each cell line was treated with the indicated dose ranges of inhibitors for 72 h, then cell viability was measured by the CellTiter-Glo assay. Each value was normalised with the value from the untreated cells. (F, G) Immunoblotting for wild-type, F2004V- or F2075C-mutated cells with ponatinib at the indicated concentrations (F; for 3 h, G; for the indicated time point). F2004V- or F2075C-mutated cells at 0 h was treated with 10 nM of cabozantinib. Cell lysates were immunoblotted to detect the indicated proteins.

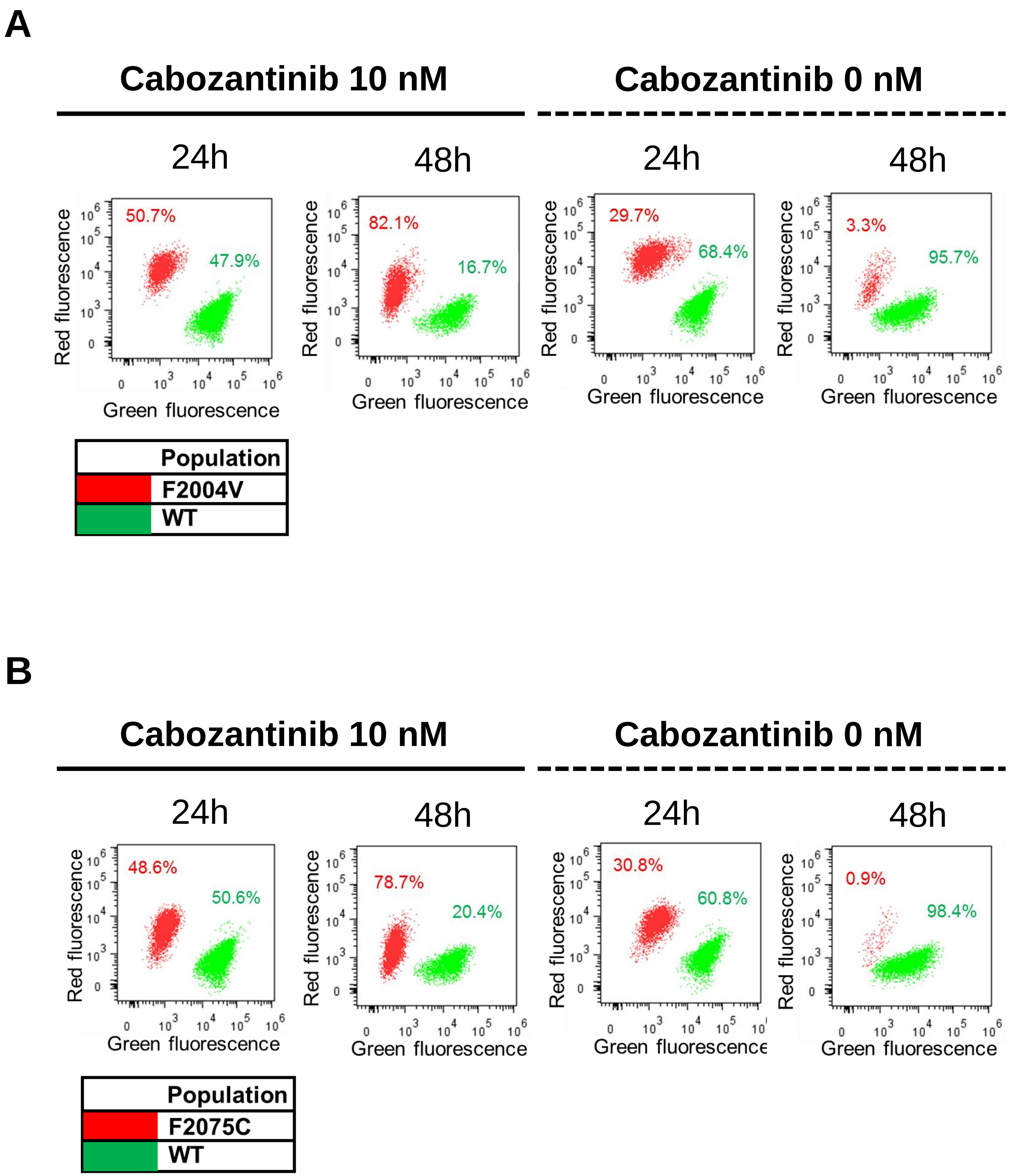

Supplementary figure S2. Dot plot of flow cytometry to evaluate proportions of cells expressing wild-type, F2004V- or F2075C-mutated CD74-ROS1.
(A, B) Dot plot of flow cytometry for cells expressing wild-type, F2004V- (A) or F2075C- (B) mutated CD74-ROS1 in the presence of 0 or 10 nM cabozantinib for 24 or 48 h. F2004V- or F2075C-mutated cells were stained with red (PKH26) and WT with green (PKH67) fluorescent dye.

A

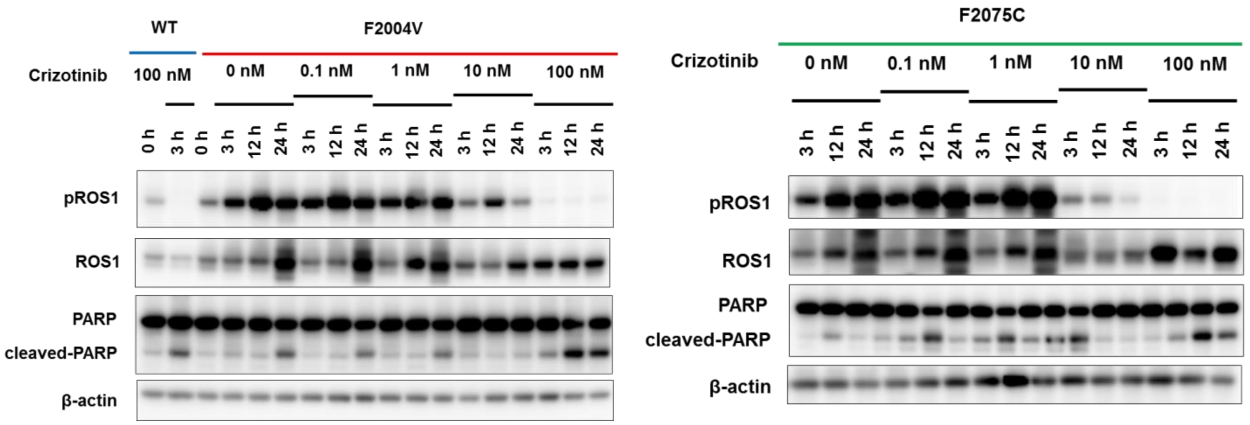

B

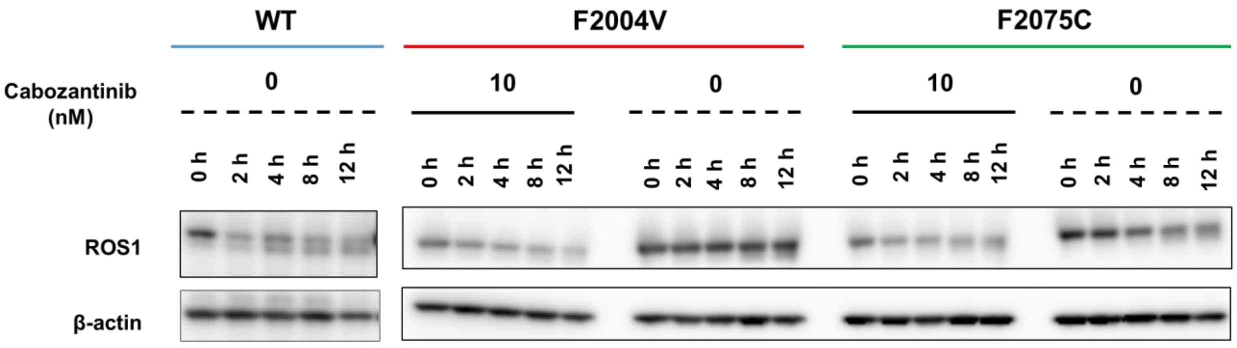

Supplementary figure S3. Crizotinib dependency and ROS1 protein stability in cells expressing wild-type, F2004V- or F2075C-mutated CD74-ROS1.

(A) Immunoblotting of cabozantinib-treated F2004V- or F2075C-mutated cells at the indicated concentrations or crizotinib-treated (100 nM) wild-type CD74-ROS1 cells. Cell lysates were immunoblotted to detect the indicated proteins. (B) Immunoblotting for untreated or cabozantinib-treated wild-type CD74-ROS1, F2004V- or F2075C-mutated cells. Each sample was treated with 100 uM of cycloheximide (CHX). F2004V- or F2075C-mutated cells were pretreated with CHX for 3 h prior to removal of cabozantinib.

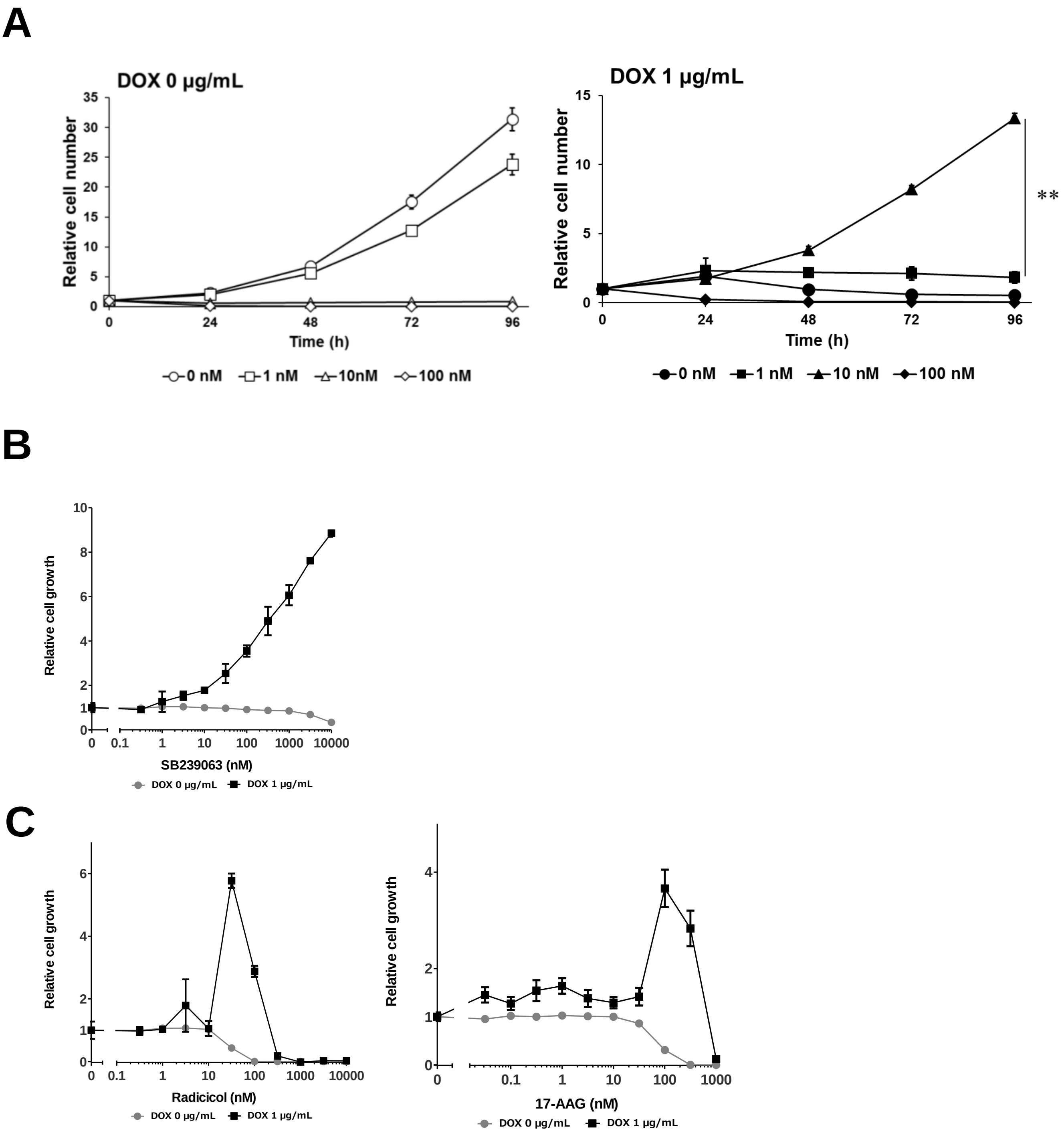

Supplementary Figure S4. Reconstructed Ba/F3 cells conditionally over-expressing F2075C-mutated CD74-ROS1 show characteristics of ROS1-TKI addiction.

(A) Cell growth of the reconstructed F2075C-mutated cells with (left) or without (right) 1 μg/mL DOX with each concentration of cabozantinib as measured by CellTiter-Glo assay. Relative cell numbers are indicated by normalizing viability of each cell line with corresponding values at 0 h. Statistical significance was calculated by a *t* test; ** indicates $p < 0.001$. (B, C) Sensitivity to SB239063 (B), radicicol (C, left) or 17-AAG (C, right) of reconstructed F2075C-mutated cells. Each the reconstructed F2075C-mutated cell lines with or without 1 μg/mL DOX was treated with the indicated concentrations of inhibitors for 72 h, then cell viability was measured by the CellTiter-Glo assay. Each value was normalised with the value from untreated cells.

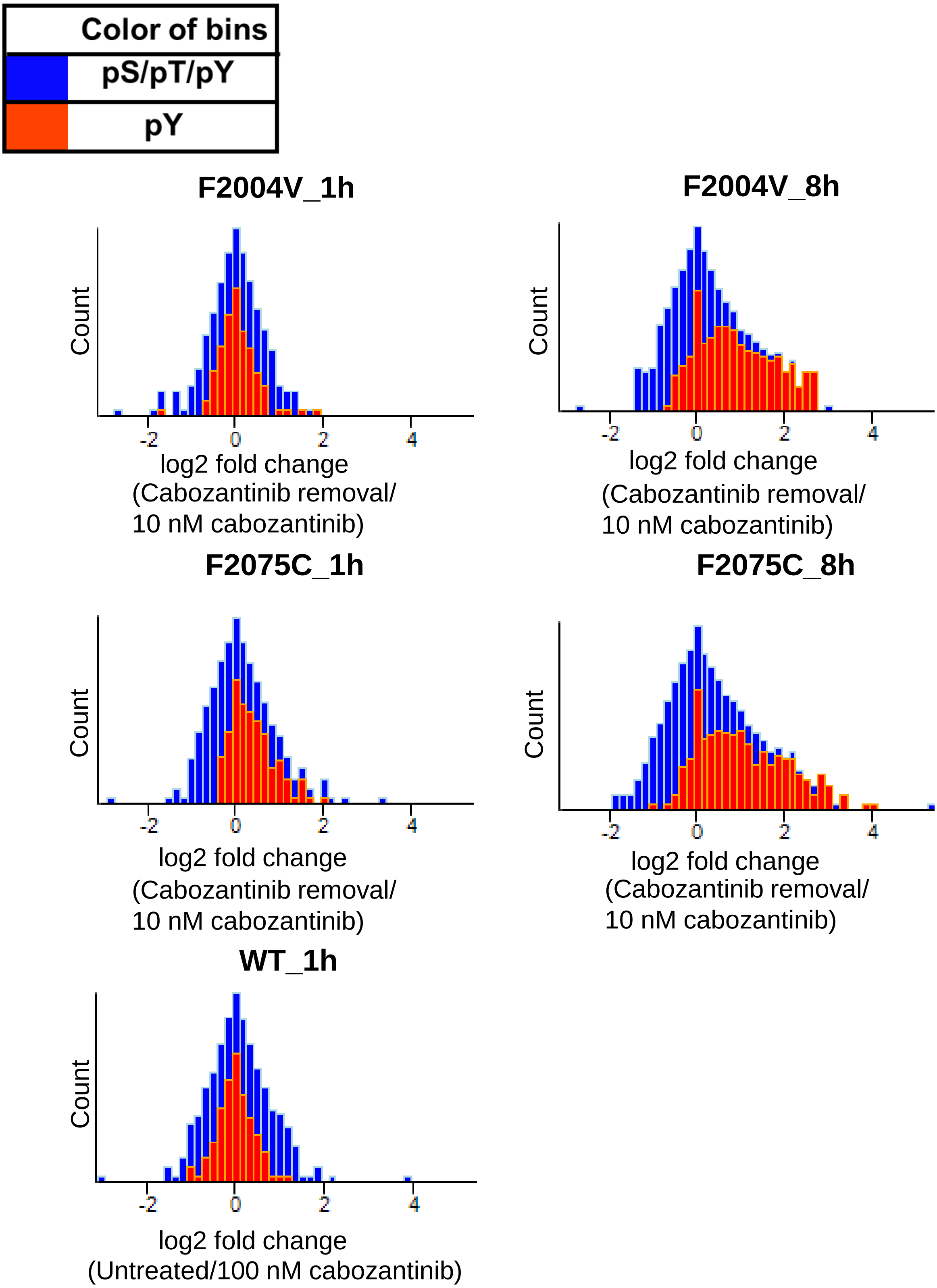

Supplementary Figure S5. Distribution of phosphorylated tyrosine peptides in histograms from phosphoproteomic analysis.
Histograms showing distributions of total peptides depicted as blue bins, and phosphorylated tyrosine (pY) peptides depicted as orange bins. Each value is shown as the mean of two independent experiments.

A

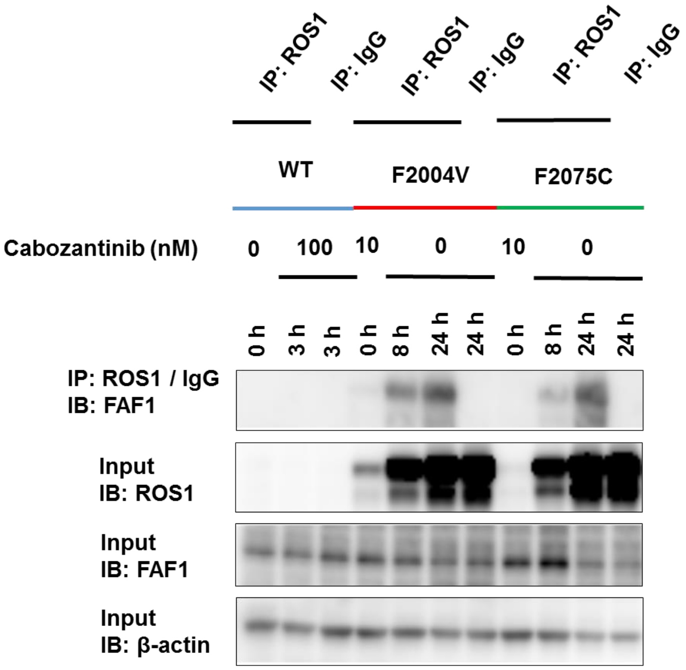

B

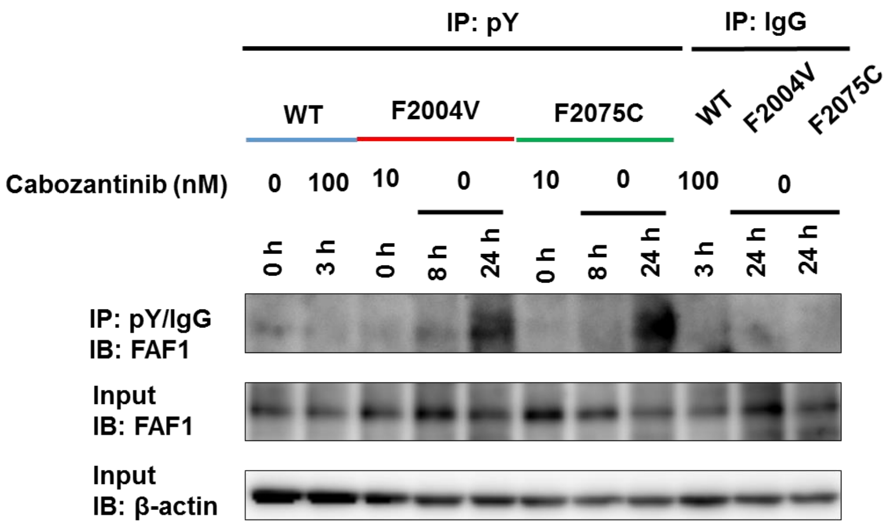

Supplementary Figure S6. ROS1-kinase phosphorylates tyrosine-phosphosites of FAF1.

(A, B) Immunoprecipitation (IP) was performed for wild-type CD74-ROS1, F2004V- or F2075C-mutated cells. The immunoprecipitated lysates were by immunoblot (IB) to detect the indicated proteins. IgG was used as a negative control. (A) Cell lysates immunoprecipitated with anti-ROS1 antibody. (B) Cell lysates immunoprecipitated with anti-phosphotyrosine antibody.

Survival	+	
	↑	
Relative cell growth (log 10)	0	
	↓	
Death	-	

		WT				F2004V				F2075C			
Target	Compound	10 nM	100 nM	1 μM	3 μM	10 nM	100 nM	1 μM	3 μM	10 nM	100 nM	1 μM	3 μM
None (control)	DMSO	0	0	0	0	0	0	0	0	0	0	0	0
antitumor (thymidylate synthetase)	5-FU	-0.0002	0.0188	-0.5533	-2.2962	-0.0644	0.0496	-0.9801	-1.9155	-0.0394	-0.0369	-0.8402	-2.2880
antitumor (aminopeptidase B)	Bestatin	0.0177	0.0457	0.0014	-0.0087	-0.0742	0.0096	-0.3118	-0.3637	-0.0573	-0.1618	-0.4646	-0.5305
antitumor (DNA)	Bleomycin sulfate	0.0289	-0.0093	-0.1122	-0.4502	-0.1407	0.0243	-0.0983	-0.3365	-0.0093	0.0567	-0.1926	-0.7023
antitumor (DNA)	Cisplatin	0.0005	0.0119	0.0628	0.0798	-0.2176	0.0157	0.0332	-0.0940	0.0246	0.0598	-0.1445	-0.0192
antitumor (DHFR)	Methotrexate	-0.0645	-0.3923	-1.0620	-1.1119	-0.1454	-0.4120	-1.3752	-1.4384	-0.0132	-0.7933	-1.7357	-1.7504
antitumor (DNA)	Mitomycin C	-0.0704	-0.3680	-2.0284	-2.3996	-0.2130	-0.0972	-0.8031	-1.3060	-0.0334	-0.1674	-1.8679	-2.3190
antitumor (tubulin)	Vinblastine sulfate	-2.1666	-2.3452	-2.4595	-2.4505	-1.7126	-2.0086	-2.1676	-2.1018	-0.1483	-2.2417	-2.5118	-2.4716
antitumor (tubulin)	Paclitaxel	-1.9705	-2.0163	-1.8658	-1.4595	-1.4957	-1.4493	-1.4600	-1.4317	-2.0970	-2.0956	-2.0933	-1.9545
antitumor (AR)	Flutamide	0.0625	0.0801	0.0764	0.1048	-0.1424	0.0563	0.0195	-0.1162	0.0299	0.0398	-0.0361	-0.0380
antitumor (DNA)	Daunorubicin, HCl	-0.3956	-2.0876	-2.2398	-2.2828	-0.2459	-1.8573	-2.1899	-2.2268	-0.0270	-2.1345	-2.3477	-2.3190
antitumor (DNA)	Doxorubicin, HCl	0.0508	-0.2034	-1.7348	-2.1582	-0.1205	-0.0992	-1.4156	-1.9692	-0.0073	-0.5063	-2.2227	-2.2002
antitumor (ER)	Tamoxifen, citrate	0.0390	0.0645	0.0942	0.0877	-0.2351	0.0119	-0.0083	-0.0916	-0.0005	-0.0830	-0.1440	-0.1483
antitumor (RNA)	Actinomycin D	-2.0126	-2.0917	-2.2243	-2.2321	-1.7762	-1.5437	-1.9372	-2.1340	-2.0186	-1.9279	-2.2807	-2.2880
antitumor (topo I)	Camptothecin	-0.0435	-0.8051	-2.2526	-2.2561	-0.2218	-0.3350	-1.7784	-2.1018	-0.0907	-0.9662	-2.3899	-2.3354
antitumor (topo I/II)	Aclarubicin	-0.0164	-2.2477	-2.3588	-2.3602	-0.3528	-1.6329	-1.5144	-2.1176	-0.0932	-2.1848	-2.4673	-2.3354
antitumor (topo II)	Etoposide (VP-16)	0.0586	0.0288	-1.6266	-2.1910	-0.2154	0.0566	-0.7749	-0.9310	-0.1312	-0.0655	-1.9370	-2.2661
actin filament	Cytochalasin D	0.0807	-0.0331	-1.3222	-1.1554	-0.1325	-0.2575	-1.6235	-1.8086	-0.0281	-0.1402	-1.9247	-2.1069
adenylcyclase	2',5'-dideoxyadenosine	-0.0171	0.0935	0.0611	0.1116	-0.1742	0.0507	-0.0295	-0.1542	-0.0465	-0.0331	-0.1275	-0.0603
AKT	AKT inhibitor	0.0806	0.0876	0.0886	0.1021	-0.1553	-0.0314	-0.0091	-0.0373	-0.0471	-0.0276	-0.1143	-0.0092
AKT	NL-71-101	0.0631	0.1021	0.0765	0.0921	-0.1691	0.0566	-0.0274	-0.1661	-0.0877	-0.0165	-0.1411	-0.0458
Bcr-Abl	AG957	0.0565	0.0770	0.0664	0.0992	-0.1516	-0.0228	-0.0257	-0.0488	-0.0900	-0.0364	-0.1058	0.0191
CAMKII	KN93	0.0593	0.0613	0.0491	-0.0456	-0.2438	-0.0252	-0.0537	0.1044	-0.0603	0.0197	-0.1734	-0.1634
caspase	Z-VAD-FMK	0.0420	0.0506	-0.0035	0.0262	-0.2299	-0.0882	-0.1620	-0.3065	-0.0510	0.0018	-0.3963	-0.5724
CDC2	Kenpaullone	0.0379	0.0549	0.0786	0.1200	-0.1689	0.0225	-0.1742	-0.2272	-0.1181	0.0252	-0.1834	-0.0575
CDK2	Purvalanol A	0.0709	0.0707	0.0841	-0.0594	-0.1319	0.0004	-0.0196	-0.3023	-0.0507	0.0023	-0.1223	-0.1049
CDK4	3-ATA	0.0471	0.0981	0.0574	-0.1066	-0.1552	0.0160	0.0844	-0.1223	-0.0697	-0.0296	-0.1168	-0.2894
CDKs	Olomoucine	0.0893	0.0930	0.1017	0.1269	-0.1839	0.0405	0.0185	-0.0844	-0.0589	0.0069	-0.0350	0.0620
CKII	TBB	0.0177	0.0753	0.1097	0.1203	-0.1633	0.0812	-0.0428	-0.1014	-0.0560	-0.0597	-0.1100	-0.0165
COX-1	Sulindac sulfide	0.0308	0.0696	0.0938	0.0978	-0.1998	0.0864	-0.0742	-0.1215	-0.0812	0.0110	-0.0555	-0.0122
COX-1	Valeryl salicylate	0.0304	0.0653	0.0956	0.1100	-0.3026	0.0278	0.0294	-0.1310	-0.0951	0.0261	-0.1908	-0.0844
COX-2	NS-398	0.0202	0.0490	0.0448	0.0525	-0.2463	-0.0572	-0.0271	-0.1043	-0.1651	0.0140	-0.2734	-0.1321
COX	Sodium salicylate	0.0036	0.0710	0.0628	0.0821	-0.2488	0.0919	0.0796	-0.0574	-0.0532	0.0501	-0.0918	-0.1220
cyclicphosphodiesterase	Theophylline	0.0851	-0.0838	0.1085	0.0597	-0.1548	0.0504	-0.0028	-0.0414	-0.0335	-0.0203	-0.0561	-0.0354
DNA methyltransferase	Azacytidine	0.0543	0.0789	-0.1169	-0.2402	-0.1581	-0.0305	-0.5115	-0.7178	-0.0766	-0.0176	-0.8463	-0.8514
DNA polymerase	Aphidicolin	0.0994	-0.0241	-1.0794	-1.7721	-0.1859	-0.0651	-0.6471	-0.7002	-0.0798	-0.1731	-1.2877	-1.3321
EGFR	AG1478	0.0736	0.1022	0.1117	0.0620	-0.1523	0.0695	0.2793	0.6027	-0.1084	0.0015	0.1747	0.6123
EGFR, topoII	Genistein	0.0384	0.0796	0.0643	0.0972	-0.2509	0.0090	-0.1054	-0.2473	-0.1501	0.0203	-0.1873	-0.2206
farnesyltransferase	Manumycin A	0.0521	0.0633	0.0596	-0.0130	-0.2631	-0.0048	-0.1425	-0.4602	-0.1246	-0.0395	-0.3717	-0.6559
farnesyltransferase	FTI-276	0.0365	0.0489	0.0450	0.0516	-0.2291	-0.0155	-0.0881	-0.1031	-0.1745	0.0390	-0.1987	-0.0934
Flk-1	SU1498	0.0791	0.0901	0.0397	0.0409	-0.1278	0.0470	0.0464	0.0435	-0.0925	-0.0190	-0.0653	-0.0074
geranylgeranyltransferase I	GGTI-286	0.0975	0.0835	0.0881	0.1114	-0.1323	0.0260	-0.0756	-0.0227	-0.1173	-0.0022	-0.0386	-0.0833
GR	Dexamethasone	-0.1407	-0.1531	-0.1478	-0.1221	-0.8654	-0.6014	-0.8392	-0.7191	-0.8717	-0.9625	-0.7979	-0.8034
GSK-3	GSK-3 inhibitor II	0.0507	0.0902	0.0975	0.1219	-0.2172	-0.0486	-0.1833	-0.2594	-0.0655	-0.0584	-0.1494	-0.3610
HDAC	Scriptaid	0.0852	0.0283	-0.8486	-1.9871	-0.1155	0.6172	-0.1639	-1.5237	0.0066	0.4282	-1.0787	-1.9615
HDAC	Trichostatin A	-0.3432	-1.8498	-1.9505	-1.9496	0.3766	-1.7760	-1.9246	-1.9579	0.3596	-1.8763	-2.1210	-2.0386
HER2 (erbB2/neu), EGFR	AG825	0.0318	0.0642	0.0802	0.1028	-0.2959	0.0388	-0.0704	-0.0850	-0.1323	0.0115	-0.1336	-0.1139
protein synthesis	Cycloheximide	0.0052	-0.2613	-0.8792	-1.5443	-0.1861	-0.0486	-0.4102	-0.6175	-0.1017	-0.0249	-0.8358	-1.2202
HMG-CoA reductase	Lovastatin	0.0355	0.0660	0.0132	-0.0779	-0.1448	0.0191	-0.0306	-0.3326	-0.1461	-0.0271	-0.1488	-0.5893
HSP90	Radicicol	0.1053	-0.9222	-2.0754	-2.0977	0.7348	-0.1538	-1.4686	-2.0175	0.1256	-0.1457	-2.1506	-2.2253
HSP90	17-AAG	0.0753	0.0842	-1.9309	-2.0166	-0.1450	0.9454	-1.1013	-1.8958	-0.0900	0.1117	-0.9718	-2.0737
IGF-1R	AG1024	0.0771	0.0713	0.1057	0.0977	-0.1698	0.0438	-0.0034	0.1080	-0.1208	0.0074	-0.0403	0.0759
iNOS	1400W, HCl	0.0273	0.0704	-0.0077	-0.0704	-0.1438	0.0515	-0.3103	-0.4317	-0.0533	-0.0085	-0.2663	-0.2563
iNOS	AMT, HCl	0.0455	0.0872	0.0854	0.1138	-0.1434	-0.0054	-0.0334	-0.1344	-0.1117	0.0208	-0.1119	-0.0586
Jak-2	AG490	0.0446	0.0655	-0.0377	-0.1280	-0.2527	0.0554	-0.1724	-0.5417	-0.1256	-0.0008	-0.3718	-0.5332
Jak-2	Cucurbitacin I	-0.2720	-0.7371	-1.0049	-1.9106	-0.6367	-0.8447	-0.6387	-1.2748	-0.3775	-0.8729	-1.2849	-1.9615
JNK	SP600125	0.0541	0.0790	0.0358	0.0627	-0.1242	0.0401	0.0768	0.0899	-0.0581	-0.0330	-0.0873	-0.0840
lck (p56), TYK	Damnacanthal	0.0952	0.0766	0.1028	0.1254	-0.1303	0.0039	0.0313	-0.0966	-0.0541	0.0002	-0.1359	-0.1939
MEK	PD 98059	0.0828	0.1285	0.1006	0.1183	-0.1664	-0.0124	-0.1489	-0.3147	-0.0899	-0.0678	-0.1877	-0.2945
MEK	U0126	0.0903	0.1007	0.0773	0.0419	-0.2138	-0.0474	-0.2814	-0.5704	-0.0669	-0.0347	-0.2353	-0.5255
methionine aminopeptidase	Fumagillin	-0.0343	-0.0765	-0.1194	-0.1224	-0.4281	-0.3085	-0.3618	-0.4051	-0.2862	-0.3025	-0.3253	-0.2878
MMP	GM 6001	-0.0348	0.0850	0.0812	0.0921	-0.2614	0.0181	-0.0611	-0.0729	-0.0473	-0.0351	-0.1208	-0.0379
NF-kB	N-Acetyl-L-cysteine	0.0668	0.0663	0.0599	0.0889	-0.1718	-0.0777	-0.1103	-0.1209	-0.0926	0.0490	-0.1633	-0.1310
NOS	Aminoguanidine, HCl	0.0387	0.0635	0.0193	0.0536	-0.2301	-0.0798	-0.0184	-0.1462	-0.0978	-0.0063	-0.2348	-0.1655
NOS	L-NMMA	0.0495	0.0540	0.0660	0.0806	-0.1651	-0.0318	-0.1182	-0.0497	-0.0425	-0.0258	-0.1927	-0.1324
p38 (MAPK)	PD169316	0.0675	0.0764	0.0232	-0.0354	-0.2008	0.1931	0.3772	0.4125	-0.0873	0.2467	0.2642	0.1824
p38 (MAPK)	SB 203580	0.0587	0.0907	0.0946	0.0585	-0.1782	0.1422	0.2365	0.2297	-0.0039	0.2265	0.2777	0.2372
p70 S6K	Rapamycin	-0.2964	-0.3332	-0.3120	-0.3616	0.1615	0.3253	0.2562	0.0857	0.0891	0.1261	0.0210	-0.0678
PARP	NU1025	0.0727	0.1108	0.1226	0.1115	-0.1791	0.0041	0.0535	-0.0196	-0.0961	-0.0279	-0.1353	0.0164
PARP-1	Benzamide	-0.0291	0.0737	0.1092	0.1242	-0.1846	-0.0002	-0.0802	-0.0784	-0.0191	-0.0086	-0.2207	-0.0435
PC-PLC	D609	0.0606	0.0744	0.0749	0.0954	-0.2801	-0.0530	0.0043	-0.0583	-0.0779	-0.0248	-0.1301	-0.0594
PDE	IBMX	0.0539	0.0730	0.0268	0.0176	-0.2480	-0.0014	-0.1194	-0.5833	-0.1410	-0.0336	-0.4140	-0.6920
PDE (cAMP)	Ro-20-1724	0.0443	0.0269	-0.1338	-0.1385	-0.1563	-0.4161	-1.2672	-1.4384	-0.1219	-0.4376	-1.0067	-1.0593
PDE (cGMP)	Zaprinast	0.1156	0.0895	0.0786	0.1032	-0.1475	-0.0039	0.0076	-0.0528	-0.1249	-0.0050	-0.1271	-0.0702
PDGFR	AG1296	0.0296	0.1117	0.0873	0.1076	-0.1909	0.0196	0.0223	-0.0793	-0.0818	-0.0245	-0.0778	-0.1183
PI3K	LY294002	0.0988	0.0826	0.0064	-0.1385	-0.1085	0.0676	0.3447	0.3060	-0.1279	0.0579	0.0977	0.1292
PI3K	Wortmannin	0.0580	0.0836	0.0853	0.0904	-0.1812	-0.0101	-0.0100	-0.0179	-0.0905	0.0016	-0.0488	-0.0385
PKA	H-89, HCl	0.0294	0.0893	0.0744	0.0569	-0.2083	0.0357	-0.0167	-0.1119	-0.1041	-0.0291	-0.1187	-0.0438
PKC	Bisindolylmaleimide I, HCl	0.0638	0.0645	-0.1424	-0.6050	-0.2428	-0.0370	-0.1096	-0.2737	-0.0788	-0.0279	-0.3377	-0.5707
PKC, PKA	H-7	0.0497	0.0410	0.0148	0.0733	-0.3388	-0.0895	-0.1479	-0.2324	-0.1880	-0.0056	-0.2328	-0.0497
PKC, PKA, PKG, MLCK	Staurosporine	-1.9341	-2.1821	-2.2408	-2.2537	-1.0671	-1.6484	-1.5602	-2.0719	-1.7464	-2.1345	-2.0231	-2.3612
PLA2	cPLA2inhibitor	0.0955	0.0956	0.0707	0.0829	-0.2132	0.0983	-0.0381	0.0771	-0.1031	-0.0840	-0.1753	-0.1540
PLA2	OBAA	0.0711	0.0984	0.0693	0.0759	-0.1320	-0.0216	0.0284	-0.0496	-0.1133	-0.0589	-0.1413	-0.0988
PP2A	Cantharidin	0.0949	0.0853	0.0592	0.0606	-0.2536	0.0480	-0.0339	-0.2735	-0.1756	-0.0228	-0.2258	-0.2479
PP2A	Cytostatin	0.0607	0.0497	-0.2932	-1.4427	-0.2252	-0.0863	-0.5538	-1.2680	-0.1147	-0.0614	-0.9462	-2.0558
PP2B/cyclophilin	Cyclosporin A	0.0783	0.1002	0.0768	0.0416	-0.2677	-0.0464	0.1364	0.1902	-0.0412	0.2025	-0.0256	-0.2740
PP2B/FKBP	FK-506	0.0926	0.0860	0.0655	0.0606	-0.4035	-0.1231	-0.1308	-0.0960	-0.0849	-0.0268	-0.1188	-0.0567
proteasome	MG-132	0.0296	0.0497	-2.1718	-2.1753	-0.2115	0.1055	-2.1464	-2.1873	-0.1425	-0.0356	-2.3397	-2.2806
proteasome	Lactacystin	0.0011	0.0315	-0.0525	-0.0139	-0.0995	0.0809	-0.0697	-0.0067	-0.1554	0.0221	-0.0828	-0.0917
ribonucleotide reductase	Hydroxyurea	0.0818	0.0710	-0.0078	0.0252	-0.2022	0.0657	-0.0179	-0.0578	-0.1920	-0.0926	-0.0666	-0.0001
ROCK	HA1077	0.0706	0.0726	0.0389	0.0644	-0.1223	0.0392	-0.1748	-0.2019	-0.2175	-0.0405	-0.0733	0.0007
ROCK	Y27632	0.0579	0.0282	0.0105	0.0281	-0.2468	-0.1435	-0.0592	-0.1600	-0.2156	-0.1180	-0.1753	-0.0773
Src, Fyn, Lck	PP1 (analog)	0.0367	0.0706	0.0114	0.0163	-0.2758	-0.0512	-0.0121	-0.0004	-0.1993	-0.0449	-0.1089	0.0452
Src, Fyn, Lck	PP-H	0.0715	0.0558	0.0209	0.0476	-0.2201	-0.0093	-0.1035	-0.1201	-0.1843	-0.1108	-0.3196	-0.2816
tubulin depolymerization	Nocodazole	0.0471	-0.5353	-2.1091	-2.1160	-0.2459	-0.9219	-1.8559	-1.8678	-0.2125	-1.1713	-2.2350	-2.2063
tyr phosphatase (PTP)	Dephostatin	0.0145	0.0069	-0.0560	0.0006	-0.1909	-0.0326	-0.1304	-0.0895	-0.1304	-0.0646	-0.1964	-0.1095

Supplementary Figure S7. High-throughput inhibitor screening for Ba/F3 cells expressing wild-type, F2004V- or F2075C-mutated CD74-ROS1.

Cell growth of wild-type CD74-ROS1, F2004V- or F2075C-mutated cells with each compound screened compared with DMSO treatment as a control is shown in a heat map with log10 values shown in each column. Red indicates that relative cell growth s is suppressed and green that relative cell growth is enhanced compared with DMSO treatment. Cells ware treated with 10, 100 nM, 1 or 3 μM of the indicated compounds for 72 h, then cell viability was measured by the CellTiter-Glo assay.

		WT				F2004V				F2075C			
Target	Compound	10 nM	100 nM	1 µM	3 µM	10 nM	100 nM	1 µM	3 µM	10 nM	100 nM	1 µM	3 µM
None (control)	DMSO	0	0	0	0	0	0	0	0	0	0	0	0
AK	ABT-702	-0.1026	-0.0031	0.0056	-0.0320	0.0075	0.0419	-0.0430	-0.0487	-0.0596	0.0741	0.0159	0.1826
AKT	Akt Inhibitor IV	-0.0958	-0.1004	-1.7464	-1.8433	-0.0197	0.1939	-1.3819	-1.9142	0.0017	0.1155	-1.8918	-1.7145
AKT	Akt Inhibitor VIII, Isozyme-Selective, Akti-1/2	-0.0509	0.0396	0.0099	-0.0425	-0.0207	0.0700	0.2131	0.4659	0.0050	0.1291	0.0109	0.3529
AKT	Akt Inhibitor XI	-0.0441	0.0492	0.0431	0.0461	-0.0140	-0.0327	-0.0382	0.0600	-0.0027	0.0853	0.1038	0.1588
AMPK	compound C	0.0169	0.0286	0.0044	-0.0160	-0.0058	0.0779	0.0234	-0.0264	0.0371	0.1359	0.0955	0.2890
ATM	ATM/ATR kinase inhibitor	0.0118	0.0534	0.0251	0.0186	-0.0137	0.0092	0.0421	0.1508	0.0216	0.1356	0.0649	0.0261
ATM	ATM kinase inhibitor	-0.0245	0.0152	-0.0036	-0.0343	-0.0109	0.0454	0.0232	0.0945	0.0226	0.0672	0.0277	0.0043
Aurora	Aurora kinase/cdk inhibitor	-0.0378	-0.0871	0.0149	-0.1080	-0.0251	-0.0318	-0.1827	-0.0640	-0.0060	0.0334	0.1157	0.3038
Aurora	Aurora kinase inhibitor II	-0.2301	-0.0026	0.0777	0.0971	-0.0284	-0.0178	-0.1475	-0.0173	-0.0744	0.1093	0.1493	0.3810
Aurora	Aurora kinase inhibitor III	-0.2002	0.0076	-0.3162	-1.7241	-0.0931	-0.0582	0.3981	-0.9365	-0.0121	0.1274	-0.3860	-1.4647
Bcr-abl	AG957	-0.1290	0.0401	0.0719	0.0674	-0.1239	-0.0223	-0.0878	0.0441	0.0096	0.0807	0.1752	0.1522
BTK	LFM-A13	-0.1113	0.0433	0.0549	0.0583	-0.0286	-0.0233	-0.0988	-0.0179	-0.0259	0.1246	0.1077	0.0560
BTK	Terreic acid	-0.0520	0.0694	0.0136	0.0064	-0.0513	-0.0473	0.0520	0.0376	0.0134	0.1446	0.0683	0.1259
CAMKII	KN-93	-0.0641	0.0603	-0.0354	-0.0684	-0.0429	-0.0284	0.0076	0.2676	0.0032	0.0988	0.0459	0.1608
CAMKII	KN-62	-0.0424	0.0292	-0.0434	-0.1054	-0.0321	0.0077	-0.1486	-0.3745	0.0685	0.0726	-0.1356	-0.3260
CAMKII	Lavendustin C	-0.0749	-0.0283	0.0455	0.0361	-0.0863	-0.0252	-0.1034	0.0042	-0.0278	0.0331	0.0531	0.0854
CDK	Kenpaullone	-0.1350	-0.0001	-0.2492	-0.5112	-0.1200	-0.0596	-0.1907	-0.0847	-0.0636	0.0884	0.0464	0.2189
CDK	purvalanol A	-0.0825	0.0331	0.0818	-0.0487	-0.0521	0.0061	-0.1411	-0.2487	-0.0463	0.1018	0.0882	0.0471
CDK	Olomoucine	-0.0750	0.0284	0.0856	0.0774	-0.1269	-0.0544	-0.0521	-0.0223	-0.0073	0.0615	0.1548	0.1743
CDK	Alsterpaullone, 2-cyanoethyl	-0.0879	-0.0283	-1.0925	-1.6424	-0.1087	-0.1808	-0.5323	-1.2411	-0.0176	0.1185	-1.0164	-1.5335
CDK	Cdk1/2 inhibitor III	-0.0205	-0.6079	-1.7360	-1.8643	-0.1375	-0.5773	-1.2344	-1.8107	0.0107	-0.4246	-1.7766	-1.8194
CDK	Cdk2/9 inhibitor	-0.0286	0.1610	-1.7392	-1.9457	-0.1131	-0.0310	-1.2558	-1.5474	0.0642	0.4724	-1.8352	-1.8617
CDK	NU6102	-0.0331	0.0635	0.0080	-0.1361	-0.1455	-0.0110	-0.2379	-0.4052	0.0209	0.0695	-0.0216	-0.0546
CDK	Cdk4 inhibitor	-0.0480	0.0367	-0.0038	-0.0450	-0.1034	-0.0022	-0.1063	-0.0035	-0.0415	0.0144	-0.2829	0.0800
CDK	NSC625987	-0.1867	-0.0043	0.0659	0.0317	-0.0749	-0.0755	-0.0567	0.0378	-0.0701	0.0588	0.0820	0.0651
Chk	SB218078	-0.1852	-1.1932	-1.5789	-1.6729	0.3673	0.5603	-1.1245	-1.5733	0.3128	-0.3083	-1.6251	-1.6315
Chk	isogranulatimide	-0.0865	0.0933	0.0679	-0.1248	-0.2080	-0.0544	-0.0147	0.0472	-0.0792	0.0513	0.1450	0.0052
Chk	Chk2 inhibitor	-0.0888	0.0792	0.0783	0.0217	-0.1516	-0.0503	-0.0981	-0.0105	-0.0823	0.0535	0.1045	0.0351
Chk	Chk2 inhibitor II	-0.0258	0.1042	0.0695	0.0517	-0.1681	-0.0439	-0.1252	-0.0639	-0.0737	0.0629	0.0539	0.0679
CK	Ellagic acid	-0.0425	0.0957	0.0708	0.0591	-0.1138	-0.0686	-0.0950	-0.0235	0.0136	0.0509	0.0967	0.0467
CK	TBB	-0.0229	0.0537	0.0221	0.0263	-0.0209	-0.0640	-0.0893	0.0234	0.0061	0.0465	0.0715	0.0673
CK	DMAT	-0.0122	0.0373	0.0750	0.0803	-0.1002	-0.0355	-0.1299	-0.2024	-0.0531	0.0495	0.0559	0.0930
CK	D4476	-0.0993	0.0679	0.1296	0.1221	-0.0816	-0.0896	0.0523	0.1498	-0.1003	0.0212	0.1807	-0.0102
Clk	TG003	-0.0519	0.0595	0.0972	0.0730	-0.1287	-0.0340	0.0404	0.2394	-0.0787	0.0385	0.1776	0.2066
DGK	Diacylglycerol kinase inhibitor II	-0.0594	0.0891	0.1032	0.1061	-0.1381	-0.1014	-0.0265	0.0466	-0.0400	0.0320	0.0943	0.1005
DNA-PK	IC60211	-0.0599	0.0751	0.0832	0.0929	-0.0985	-0.0949	-0.0845	0.0048	-0.0780	0.0269	0.1438	0.0567
eEF2	TX-1918	-0.0169	0.1011	0.0625	0.0869	-0.1298	0.0078	0.0083	0.1782	-0.0320	0.0495	0.0939	0.2184
EGFR	BPIQ-Ⅱ	-0.0358	0.0793	0.0540	0.0489	-0.1171	-0.0441	-0.1284	0.1552	-0.0137	0.0532	0.0343	0.1652
EGFR	AG1478	-0.0178	0.0626	0.0328	0.0018	-0.0978	-0.0432	0.0699	0.5568	-0.0140	0.0385	0.1923	0.7000
EGFR	AG490	-0.0730	-0.0541	0.0132	-0.1145	-0.1096	-0.0148	-0.0981	-0.2314	-0.0686	0.0754	0.0067	0.0569
FGFR	SU4984	-0.1614	-0.0170	0.0942	0.0745	-0.1569	-0.1235	-0.0311	0.1624	-0.0704	0.0628	0.1082	0.1589
FGFR	SU5402	-0.1019	0.0469	0.0805	0.0810	-0.1924	-0.0685	-0.0471	-0.0412	-0.0824	0.0291	0.0977	-0.0167
Flt-3	Flt-3 Inhibitor	-0.1062	0.0378	0.0573	-0.0066	-0.1604	-0.1276	0.0308	0.2670	-0.0670	0.0087	0.1590	0.4277
Fms	cFMS Receptor Tyrosine Kinase Inhibitor	-0.1151	0.0483	0.0738	-0.0655	-0.1630	-0.0716	-0.0023	0.1918	-0.0687	0.0181	0.1198	0.1097
Fyn	SU6656	-0.0302	0.1041	0.0427	-1.5867	-0.0892	-0.0077	0.2357	0.2625	-0.0198	0.1300	0.3352	-0.9020
GSK	GSK-3 inhibitor IX	-0.0462	0.0817	-0.1805	-1.0627	-0.1874	-0.0928	-0.2633	-0.3411	-0.0239	0.0548	-0.0308	-0.4191
GSK	1-Azakenpaullone	-0.0303	0.0524	-0.3896	-1.0016	-0.1014	-0.0861	-0.2093	-0.2546	-0.0138	0.0312	-0.0526	-0.2834
GSK	indirubin-3'-monoxime	-0.0877	0.0123	0.0358	-0.4560	-0.0681	-0.0458	-0.1410	-0.1369	-0.0402	0.0270	0.0186	-0.1410
HER2	AG825	-0.0395	-0.0118	0.0700	0.0567	-0.1597	-0.1611	-0.0536	0.0094	-0.0828	0.0503	0.0913	0.1199
IGF-IR	AG1024	-0.0033	0.0516	0.1047	0.0882	-0.1535	-0.1052	-0.0199	0.1566	-0.1194	0.0057	0.1074	0.2066
IGF-IR	AGL 2263	-0.0242	0.0464	0.1079	0.0966	-0.2281	-0.1061	-0.0721	-0.0297	-0.1076	0.0042	0.1044	0.0554
IKK	BMS-345541	-0.0080	0.0755	0.0927	-1.2247	-0.2456	-0.1612	0.0345	-0.3027	-0.0828	0.0323	0.3092	-0.4717
IKK	IKK-2 inhibitor VI	-0.0140	0.0278	-1.2607	-1.2810	-0.2118	-0.1622	-0.4272	-0.7127	-0.0830	-0.0249	-0.7520	-1.1386
IRAK	IRAK-1/4 inhibitor	-0.0104	0.0826	0.0389	0.0397	-0.2147	-0.0795	-0.0301	0.0423	-0.0382	0.0732	0.0591	0.2137
Jak	JAK Inhibitor I	-0.0022	-0.0025	-0.3098	-0.6726	-0.1593	-0.0511	0.3038	0.2188	-0.0497	0.0628	0.0510	0.2592
Jak	JAK3 Inhibitor VI	-0.1709	-0.1286	-1.0418	-1.8982	-0.0642	-0.2133	-1.3270	-1.7914	-0.0146	-0.0348	-0.6433	-1.8434
JNK	SP600125	-0.1506	0.0111	0.0496	0.0370	-0.1724	-0.0379	-0.0174	0.1481	-0.0572	0.0374	0.0930	0.2097
JNK	JNK inhibitor VIII	-0.0594	0.0274	0.0927	0.1140	-0.0791	-0.0501	0.0079	0.1809	-0.0489	0.0594	0.1293	0.4469
Lck	Damnacanthal	-0.0650	0.0745	0.0838	0.0858	-0.1666	-0.0720	-0.1094	-0.0279	-0.0607	0.0204	0.0783	0.0203
Lck	PP2	-0.0704	0.0665	-0.0661	-0.4280	-0.2023	-0.0516	-0.1871	-0.2427	-0.0726	0.0337	0.0380	0.0387
MAPK	ERK inhibitor II	-0.0533	0.0917	0.0598	0.0778	-0.2182	-0.1380	-0.0235	0.1715	-0.0495	0.0706	0.1585	0.3617
MEK	PD98059	-0.0459	0.0775	0.0076	-0.0781	-0.1787	-0.1178	-0.1846	-0.2992	-0.0375	0.0348	0.0219	-0.1059
MEK	U-0126	-0.0319	0.0516	-0.0918	-0.2531	-0.1608	-0.0375	-0.3502	-0.5884	-0.0184	-0.0138	-0.0869	-0.1623
MEK	MEK inhibitor I	-0.2809	0.0059	-0.0285	-0.0298	-0.0899	-0.0447	-0.0909	-0.0009	-0.0506	0.0603	0.1005	0.1454
Met	SU11274	-0.0669	0.0577	0.0198	-0.4994	-0.1413	-0.0360	0.4130	0.5135	-0.0583	0.0849	0.1772	0.1084
MLCK	ML-7	-0.0755	0.0650	0.0826	0.0870	-0.1727	-0.1364	-0.0900	-0.0086	-0.0540	0.0148	0.0502	0.0583
p38	SB202190	-0.1361	0.0782	0.0773	0.0424	-0.1049	0.0283	0.1880	0.3110	-0.0339	0.2950	0.2558	0.6553
p38	SB239063	-0.1327	0.1095	0.0729	0.0570	-0.0982	0.0427	0.0626	0.2246	-0.0428	0.3003	0.2507	0.5753
PDGFR	AG1296	-0.0824	0.0982	0.0552	0.0711	-0.2119	-0.0886	-0.0359	0.0177	-0.0419	0.0624	0.1246	0.0506
PDGFR	SU11652	-0.0057	0.1064	-0.5635	-1.6833	-0.1933	0.1172	0.6913	-1.4841	0.0032	0.2788	-0.2178	-1.6087
PDGFR	PDGF receptor tyrosine kinase inhibitor V	-0.0074	0.0576	-0.2990	-1.5478	-0.1720	0.0518	0.6561	0.1853	-0.0352	0.0439	0.2741	0.3192
PDGFR	PDGF receptor tyrosine kinase inhibitor IV	-0.1902	-1.9262	-1.8753	-1.9606	-0.1600	-0.6271	-1.6501	-1.7977	-0.0719	-1.7691	-1.6960	-1.9137
PI3K	LY-294002	-0.0895	0.0338	-0.0025	-0.1656	-0.2169	-0.0814	0.1170	0.3291	-0.0416	0.0426	0.0905	0.3837
PI3K	Wortmannin	-0.0133	0.0475	0.0878	0.0745	-0.1682	-0.0922	-0.0923	-0.0289	-0.0815	-0.0176	0.0846	0.1087
PKA	H-89	-0.0359	0.0551	0.0916	0.0419	-0.0953	-0.0801	-0.1156	-0.0730	-0.0438	-0.0121	0.1074	0.0914
PKA	4-cyano-3-methylisoquinoline	-0.0593	0.0800	0.0904	0.0926	-0.1922	-0.1469	-0.0776	0.0055	-0.0525	0.0338	0.1304	0.0667
PKC	Bisindolymaleimide I, HCl	-0.0207	0.1073	-0.0429	-0.3296	-0.2060	-0.0821	-0.0735	0.1163	-0.0029	0.0898	-0.1785	-0.0938
PKC	Go7874	-0.0477	-0.0581	-1.5774	-1.7817	-0.1262	0.5641	-0.2407	-1.6216	-0.0262	0.3633	-1.6623	-1.7438
PKG	Rp-8-CPT-cGMPS	0.0043	0.0440	0.0151	0.0213	-0.1773	-0.0546	-0.1115	-0.0420	-0.0409	0.0176	0.0112	0.0320
PKG	KT5823	-0.2137	-0.0141	-0.0732	-0.3064	-0.1238	-0.0376	0.0767	0.6904	-0.0768	0.0105	0.1135	0.6519
PKR	PKR inhibitor	-0.9654	-1.4332	-1.5672	-1.7760	-0.4765	-0.4722	-0.7533	-0.8180	-0.4132	-0.6038	-0.7838	-1.1112
Raf	RAF1 kinase inhibitor I	-0.0239	0.0230	0.0756	0.0716	-0.1129	-0.1531	-0.1637	-0.0589	-0.0690	-0.0275	0.1108	0.1116
Raf	ZM 336372	-0.0819	0.0694	0.0916	0.0441	-0.1740	-0.0729	0.0371	0.3255	-0.0814	0.0763	0.2394	0.7233
ROCK	H-1152	-0.0340	0.0492	-0.4220	-1.5945	-0.1394	-0.0096	0.8280	0.0259	-0.0352	0.2314	0.4386	-0.7801
ROCK	Y-27632	-0.0125	0.0720	0.0141	0.0036	-0.2859	-0.1316	-0.1421	-0.0117	-0.0526	0.0366	0.0695	0.1239
Hsp90	radicicol	-0.0129	-0.1825	-1.5885	-1.7895	-0.0332	1.2629	-1.3893	-1.5928	-0.0275	0.4211	-1.5963	-1.7212
Src	PP1 analog	0.0121	0.0293	0.0146	-0.0147	-0.1700	-0.0168	-0.1115	0.1062	-0.0393	0.0323	0.1054	0.2615
Syk	Syk inhibitor	-0.1876	-0.0476	-0.0134	-0.0690	-0.1621	-0.0128	-0.0812	0.0541	-0.0938	-0.0486	-0.0106	0.0936
TGF-βRI	SB431542	-0.0461	0.0312	0.0681	0.0077	-0.1211	-0.1037	-0.0684	0.1131	-0.0840	-0.0015	0.1114	0.1601
TGF-βRI	TGF-b RI kinase inhibitor II	-0.0454	0.0116	0.0661	0.0659	-0.2507	-0.2144	-0.3888	-0.2984	-0.0333	0.0260	-0.1088	-0.1281
Tpl2	Tpl2 kinase inhibitor	-0.0691	0.0291	0.0551	0.0056	-0.1669	-0.0570	-0.1544	0.0227	-0.0440	0.0286	-0.0117	0.1846
TrkA	TrkA inhibitor	-0.0065	0.0473	-0.0341	-0.1802	-0.1308	-0.1005	0.1628	0.8604	-0.0620	0.0681	0.0822	0.3421
VEGFR	VEGFR receptor tyrosine kinase inhibitor II	-0.0122	0.0514	0.0255	0.0092	-0.1518	-0.1729	-0.1843	0.1327	0.0079	0.0295	-0.0226	0.1682
VEGFR	VEGF recptor 2 kinase inhibitor I	-0.0642	0.0298	0.0351	0.0242	-0.1624	-0.1283	-0.1374	-0.0358	-0.0228	0.0758	-0.0292	0.0722
VEGFR	SU1498	-0.0202	0.0090	-0.0055	-0.0147	-0.1582	-0.0420	-0.1409	0.1091	-0.0778	-0.0220	-0.0489	0.0940

Supplementary Figure S7. Continued

		WT				F2004V				F2075C			
Target	Compound	10 nM	100 nM	1 µM	3 µM	10 nM	100 nM	1 µM	3 µM	10 nM	100 nM	1 µM	3 µM
None (control)	DMSO	0	0	0	0	0	0	0	0	0	0	0	0
ALK	ceritinib	-0.1955	-1.4999	-2.4379	-2.5039	0.6010	-0.6357	-2.2133	-2.3622	0.8743	-1.0651	-2.1863	-2.2583
ALK	ASP3026	-0.0839	-0.5690	-2.1589	-2.4798	0.2238	0.7355	-1.4116	-2.2239	0.3761	0.7332	-1.8698	-2.1951
ALK	TAE684	-0.3981	-0.6115	-2.3406	-2.4481	0.6800	0.0375	-2.1361	-2.3845	0.8396	-0.0699	-2.1186	-2.2231
IGF1R	AEW541	0.0592	0.0471	-0.0664	-2.4352	0.0690	0.0414	-0.9236	-2.2741	0.0529	-0.0610	-0.4416	-2.1818
ALK	AP26113-analog	-0.1584	-0.1520	-0.4448	-2.4547	0.6198	0.7564	-2.1068	-2.3308	0.9066	0.8322	-2.0562	-2.2136
ALK/MET/VEGFR/RET	cabozantinib	-0.7475	-1.8916	-2.0951	-2.3713	0.5965	-0.8606	-1.9413	-2.1934	0.6402	-0.7312	-2.0050	-2.2136
VEGFR	E7080	-0.0037	-0.0298	-0.0421	-0.0952	0.0131	0.1136	0.1993	0.2661	0.0393	0.0516	0.0221	0.1505
Bcr-Abl	nilotinib	0.0897	0.0665	0.0512	-0.0451	-0.0751	0.1338	0.3573	0.0959	0.0608	0.1115	0.3010	-0.0430
BRAF/KIT/VEGFR/PDGFR	sorafenib	0.0099	0.0087	0.0438	-0.2323	0.0359	0.2307	0.4792	0.2969	0.0252	0.2330	0.5720	0.6433
mTOR	temsirolimus	-0.3173	-0.3581	-0.3840	-0.4008	0.0072	-0.0393	0.0175	-0.1028	0.2699	0.1918	0.2285	0.0990
EGFR/Her2	lapatinib	0.0510	0.0365	0.0636	0.0501	0.0062	0.0317	0.0482	-0.1677	0.0083	-0.0009	0.0464	0.0623
Bcr-Abl/Kit	imatinib mesylate	0.0148	0.0463	0.0647	0.0272	0.0293	0.0563	0.1302	0.1930	-0.0019	-0.0720	0.0862	0.1037
RET/KIT/VEGFR/PDGFR	sunitinib malate	0.0772	-0.0637	-0.4547	-2.2425	-0.0331	0.1265	0.4036	-1.5859	-0.0110	0.1632	0.5275	0.0065
EGFR	gefitinib	0.0576	-0.0580	-0.0477	-0.1158	-0.0073	0.0296	-0.0997	-0.2765	-0.0438	-0.0826	-0.1994	-0.6257
HDAC	vorinostat	0.0140	-0.0365	-0.3187	-1.6599	0.0368	0.1053	-0.0675	-1.7138	0.0116	0.1376	0.1234	-1.1956
EGFR	erlotinib	0.0752	0.0831	0.0438	0.0301	-0.0024	-0.0044	0.1237	0.2422	-0.0275	-0.0052	0.3020	0.3668
Proteasome	bortezomib	-1.9879	-2.0474	-2.1756	-2.2452	-1.8432	-1.8450	-1.9914	-2.0892	-1.7321	-1.8971	-1.9914	-2.0071
Bcr-Abl/Src	dasatinib	0.1333	0.0471	-0.4649	-0.6529	-0.0134	-0.1139	-0.7941	-1.0761	-0.0551	-0.3906	-1.1133	-1.6011
mTOR	everolimus	-0.2093	-0.2999	-0.3762	-0.3980	0.0555	-0.0420	-0.0028	-0.1411	0.2849	0.1318	0.1958	0.0867
FGFR/KIT/VEGFR/PDGFR	pazopanib	0.1084	0.0944	-0.3346	-1.0261	0.0279	0.0911	0.7003	0.1910	0.0017	0.0971	0.9402	0.3486
Rho/SRF	CCG-1423	0.1617	0.0699	0.0913	0.0699	-0.0019	0.0128	0.0248	0.0558	-0.0205	-0.0743	0.0106	0.0147
PIM	PIM1/2 Kinase Inhibitor V	0.0941	0.0014	0.0302	0.0397	0.0245	0.0243	-0.0104	0.0217	-0.0201	-0.0745	0.0306	0.0940
PIM	PIM1 Inhibitor II	0.0710	0.0002	0.0523	0.0356	-0.0166	0.0338	0.0347	-0.0455	-0.0189	-0.0114	0.0425	0.0472
Hedgehog	AY 9944	0.1363	0.1170	0.0731	0.0520	-0.0243	-0.0227	-0.0629	-0.2532	-0.0367	-0.0920	-0.0170	-0.1474
Hedgehog	cyclopamine	0.1804	0.1171	0.0988	0.0709	0.0155	0.0117	0.0781	-0.0692	-0.0490	-0.0540	0.0351	-0.0091
Hedgehog	Jervine	0.0877	0.1658	0.1135	0.0920	0.0066	-0.0217	0.1225	0.0235	-0.0270	-0.0747	0.0269	0.0154
STAT3	WP1066	0.1616	0.1448	0.0357	-0.5824	-0.0097	-0.0173	-0.0329	-0.5717	-0.0834	-0.0699	0.1943	-0.2797
STAT3	5,15-DPP	0.1710	0.1430	0.1060	0.0806	0.0108	-0.0146	0.1071	0.0534	-0.0430	-0.1093	0.0566	-0.0051
Wnt	IWP-2	0.1641	0.1082	0.0889	0.0691	-0.0167	0.0174	0.0950	0.0532	-0.0594	-0.0204	0.0562	0.0108
Wnt	IWR-1-endo	0.0563	0.0445	0.0166	-0.0262	0.0369	0.0232	0.0246	0.0363	-0.0411	-0.0780	0.0845	0.1970
Wnt	FH535	0.0505	0.0071	0.0573	0.0349	-0.0298	0.0986	0.0667	0.0119	-0.0478	-0.0933	0.0197	0.0593
Notch	DAPT	0.1086	0.1283	0.0990	0.0951	-0.0009	0.0158	0.0535	-0.0017	-0.0477	-0.1314	-0.0445	-0.0711
tankyrase-selective PARP	XAV939	0.1662	0.1392	0.1322	0.1263	-0.0493	0.0940	0.0926	0.0437	-0.1205	-0.1328	0.0385	0.0661
pan-PARP	PJ-34	0.1577	0.1585	0.1096	0.0806	0.0425	-0.0152	0.0382	0.0211	-0.0963	-0.1558	0.0156	0.0408
PARP-1/2-selective	Olaparib	0.1359	0.1413	-0.0073	-0.4014	0.0070	-0.0611	-0.0254	-0.6724	-0.0856	-0.1410	0.1002	-0.2553
antipsychotic drug	chlorpromazine hydrochloride	0.1588	0.1508	0.1156	0.0999	0.0330	-0.0630	0.0487	-0.1171	-0.0748	-0.1728	0.0053	0.0215
depression treatment	desipramine hydrochloride	0.1672	0.1238	0.0985	0.0763	-0.0193	-0.0518	0.0345	0.0669	-0.0847	-0.1377	0.0110	0.1167
golgi inhibitor	brefeldin A	0.1141	0.0607	-0.0280	-2.1888	0.0581	-0.0001	-0.4521	-2.0892	-0.0797	-0.1965	-0.5775	-2.0634
stress inducer	anisomycin	0.0313	-1.2519	-1.7417	-2.0716	0.0444	-1.6173	-1.0112	-1.4116	0.0214	-1.2895	-0.6300	-0.9195
thalidomide family	thalidomide	0.1024	0.0878	0.1090	0.0922	0.0467	0.0137	0.0711	-0.0203	-0.0061	-0.0832	0.0191	0.0091
thalidomide family	lenalidomide	0.0815	0.0717	0.1403	0.1272	0.0267	0.0192	0.1025	0.0211	-0.0190	-0.1180	0.0516	-0.0113
retinoids	tretinoin	0.0672	0.0594	-0.0040	-0.1072	-0.0858	-0.1922	-0.3134	-0.5945	-0.2954	-0.4003	-0.4511	-0.4932
retinoids	tamibarotene	0.0484	0.0771	0.0810	0.0845	-0.0734	-0.1172	0.0021	-0.0661	-0.2473	-0.3861	-0.3209	-0.3080
DNA alkylation	temozolomide	0.0771	0.1165	0.1149	0.1182	0.0322	-0.0401	0.0094	-0.0094	-0.0099	-0.0964	0.0107	-0.1017
EML4-ALK	crizotinib	-0.5117	-1.4339	-1.9790	-2.0851	0.5816	-0.9702	-1.9082	-2.0558	0.9080	-0.9053	-1.8382	-1.9815
mTOR	Torkinib	0.1052	0.0104	-0.6637	-1.8917	0.0196	0.0603	-0.3196	-1.5733	-0.0482	-0.0433	-0.0492	-1.4162
lipase	orlistat	0.0431	0.0232	0.0436	0.0139	0.0277	0.0474	0.0413	-0.0574	-0.0574	-0.1173	-0.0057	0.0224
AR	MDV3100	0.1397	0.1002	0.1040	0.0810	-0.0380	0.0158	0.0829	-0.0314	-0.0568	-0.1027	0.0735	0.0326
caspase activator	PAC-1	0.1000	0.1053	0.0659	-0.3860	-0.0345	0.0186	-0.2168	-0.8533	-0.0865	-0.1028	-0.4392	-0.8170
blc-2	ABT-737	0.1254	0.1258	-0.0188	-0.1876	0.0246	-0.0224	-0.1247	-0.3598	-0.0766	-0.1208	0.0771	0.0121
G9a	UNC0638	0.1204	0.1274	-0.7301	-2.0832	0.0796	0.0913	-1.3870	-2.0400	0.2234	0.2807	0.0515	-2.0344
G9a	BIX01294	0.1019	0.1484	-1.1222	-2.0484	0.0165	0.0587	-1.5512	-2.0247	-0.0389	0.0398	-0.3857	-1.9625
LSD1	S2101 (LSD1 inhibitor II)	0.1446	0.0900	0.0916	0.0859	0.0070	-0.0496	0.0027	-0.1483	-0.0938	-0.0987	-0.0804	-0.0765
PRMT1	AMI-1	0.1180	0.0250	0.0467	0.0358	0.0449	0.0157	-0.0096	-0.0687	-0.0550	-0.0567	0.0021	0.0160
p300	C646	0.1005	0.0424	0.0542	0.0286	-0.0488	0.0355	-0.0075	-0.0549	-0.0192	-0.0582	0.0177	0.0647
SIRT1	SIRT1 inhibitor III	0.1418	0.1252	0.0835	0.0625	-0.0309	0.0073	0.0906	0.0107	-0.0668	-0.0497	0.0436	-0.0259
SIRT1/2	Tenovin-6	0.0876	0.1368	-1.0231	-2.1526	0.0443	0.0006	-1.5530	-2.0778	-0.0764	-0.1575	-1.2127	-2.0875
HDAC8	PCI-34051	0.1616	0.1570	0.1099	0.0374	0.0119	-0.0387	0.0363	-0.1274	-0.0766	-0.1172	0.0644	-0.2538
BRD4 bromodomain	(+)-JQ1	0.0932	-0.4849	-1.5069	-1.9877	0.1442	-0.4247	-1.6832	-1.9911	0.1288	-0.0819	-0.7092	-1.4715
Telomerase	TMPyP4	0.1505	0.1592	0.0994	0.0118	0.0659	0.0383	0.1024	0.0924	-0.0544	-0.0699	0.1937	0.1834
PARP	BSI-201 (Iniparib)	0.1798	0.1212	0.0626	0.0282	0.0566	-0.0179	0.1159	0.1519	-0.0106	-0.0561	0.1549	0.2587
PARP	ABT-888 (Veliparib)	0.1184	0.0371	0.0031	-0.0209	0.0061	-0.0054	-0.0054	-0.0076	-0.0777	-0.0668	0.0129	-0.0478
PARP	AG014699 (Rucaparib)	0.0563	-0.0176	-0.2301	-0.5583	-0.0681	-0.1177	-0.4575	-0.8909	-0.0061	-0.0969	-0.1882	-0.4288
PARP	MK-4827 (Niraparib)	0.1243	0.0626	-0.3455	-1.1020	0.0121	-0.0362	-0.5232	-1.4497	-0.1238	-0.1326	-0.1457	-0.6654
Aurora	ENMD-2076	0.1130	0.0984	-1.4930	-2.2232	-0.0273	0.1839	-0.1116	-2.1009	-0.0880	0.0260	-0.0800	-1.5449
Aurora	MLN8237	0.1048	-0.3164	-2.0838	-2.1677	0.0397	-0.3375	-1.8027	-1.9775	-0.0264	-0.1136	-0.9002	-1.4647
Survivin	YM155	0.0965	0.0285	-2.0624	-2.1706	0.0388	-0.5400	-1.9634	-2.0722	-0.0640	-0.4773	-1.7852	-2.0221
PDK1	OSU-03012	0.1177	0.1344	0.1089	0.0931	0.0872	0.0508	0.0586	0.0132	-0.0323	-0.1523	0.0607	-0.0216
IGF-IR	OSI-906	0.1313	0.0727	0.0132	-0.0214	-0.0037	-0.0019	-0.0530	-0.1882	-0.1014	-0.0837	0.0677	-0.1536
c-Met	PF-04217903	0.1070	0.0290	0.0187	0.0036	0.0763	0.0337	0.0438	-0.0163	-0.0583	-0.0667	0.0599	0.0873
DNMT	Decitabine	0.0394	-0.1196	-0.8066	-1.7827	0.0174	-0.1241	-1.2173	-1.8150	-0.0106	-0.1509	-0.9375	-1.3971
VEGFR/EGFR/RET	Vandetanib	0.1095	0.0860	0.0551	-0.0932	0.0192	0.0835	0.3258	0.3597	-0.0663	-0.1205	0.0996	0.1261
VEGFR/PDGFR/KIT	Axitinib	0.1167	0.0303	-0.8260	-2.2959	0.0627	0.1607	0.4450	-2.0005	-0.0762	0.0239	0.4341	-1.0712
BRAF	Vemurafenib	0.1001	0.0569	0.0803	0.0549	0.0623	0.1370	0.3810	0.3371	-0.1020	-0.0247	0.3436	0.3383
JAK	Ruxolitinib	0.0528	0.0303	-0.0292	-0.1734	0.0582	0.0888	0.4498	0.5203	-0.1046	-0.1514	0.2823	0.4266
Hedgehog	Vismodegib	0.0613	0.0805	0.0925	0.0830	0.0946	-0.0073	0.0898	0.0883	-0.0496	-0.0997	0.0644	0.0422
GLI1	Gant61	0.0745	0.0017	0.0336	-0.0810	0.0522	0.0070	-0.0066	-0.3828	-0.0491	-0.1157	0.0021	-0.2592
FGFR	PD173074	0.0466	-0.0012	-0.0628	-0.1382	0.0897	0.0850	-0.0764	-0.5252	-0.0707	0.0101	0.1155	-0.3299
ALK	A83-01	0.0282	0.0233	0.0099	-0.0164	0.0191	0.0016	0.0270	0.0250	-0.1073	-0.3119	-0.1909	-0.1830
GSK-3	BIO	0.0852	0.0966	-1.2703	-1.9183	-0.0550	-0.1000	-0.9496	-2.0005	-0.0759	-0.2728	-0.7476	-1.5118
GSK-3	TWS119	0.1004	0.0763	0.0718	-0.0522	0.0264	0.0012	0.3342	0.4928	-0.0552	-0.1234	0.2591	0.2391
GSK-3	CT99021	0.0935	0.0830	0.1005	-0.3399	0.0712	-0.0191	-0.2874	-0.7267	-0.0822	-0.1484	-0.2488	-0.5208
TGFb-R	LY2157299	0.0801	0.0988	0.0575	0.0179	0.0800	0.0550	0.1394	0.0945	-0.0701	-0.1827	-0.0069	-0.0287
TGFb-R	SD208	0.0761	0.1108	0.0359	-0.0439	0.0055	-0.0401	0.0092	-0.0755	-0.1226	-0.2551	-0.1616	-0.0771
ALK	LDN193189	0.1456	0.0714	-0.3044	-2.1592	0.0052	0.1403	-0.3964	-2.1130	-0.0334	-0.1118	-0.5585	-2.0875
ROCK	Thiazovivin	0.0913	0.0301	-0.0036	-0.0364	0.0393	0.0666	-0.0272	-0.0330	-0.1188	-0.1269	-0.1182	-0.2341
VEGFR etc.	motesanib	0.0543	-0.0007	-0.0322	-0.0954	-0.0391	0.0047	0.3099	0.4167	-0.0588	-0.1087	0.2969	0.5370
NTRK/FLT3	CEP701	-0.7155	-2.0981	-2.3033	-2.4352	0.3638	-2.0229	-2.0649	-2.1649	0.7472	-1.8482	-2.0298	-2.1562
MET/VEGFR	foretinib	-0.7040	-1.8774	-2.2703	-2.3569	0.2375	-1.6372	-2.0162	-2.0558	0.3552	-1.5939	-2.0485	-2.1243
BCR-ABL	Ponatinib	0.0304	-0.1543	-2.1957	-2.3092	0.4003	0.5988	-2.0214	-2.0950	0.2596	0.7855	-2.0085	-2.1321
EGFR/HER2	Afatinib	0.0170	0.0196	-0.2649	-2.0462	0.0347	0.0644	-0.3942	-2.1719	-0.1610	0.0082	0.2920	-2.1951
VEGFR/PDGFR/FGFR	nintedanib	0.0435	-0.0330	-0.6341	-1.9794	0.0392	0.0277	-0.9086	-1.9911	-0.1517	-0.0600	-0.1616	-0.4522

Supplementary Figure S7. Continued

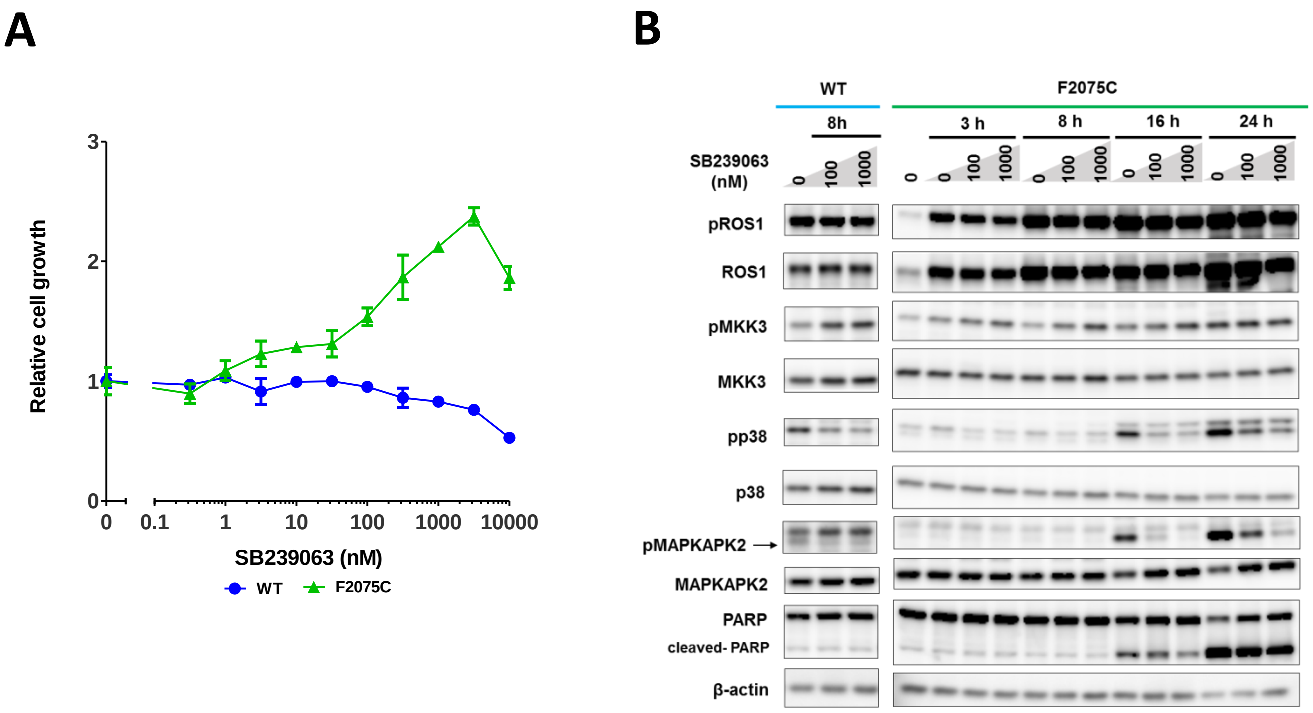

Supplementary Figure S8. Rescue effect of p38 inhibitor (SB239063) on cells expressing F2075C-mutated CD74-ROS1.

(A) Sensitivity to SB239063 of wild-type (WT) or F2075C-mutated cells. Each cell line was treated with the indicated dose ranges of inhibitors for 72 h, then cell viability was measured by the CellTiter-Glo assay. Each value was normalised with the value from untreated cells.

(B) Immunoblot for WT or F2075C-mutated cells treated with SB239063. Cell lysates were immunoblotted to detect the indicated proteins.

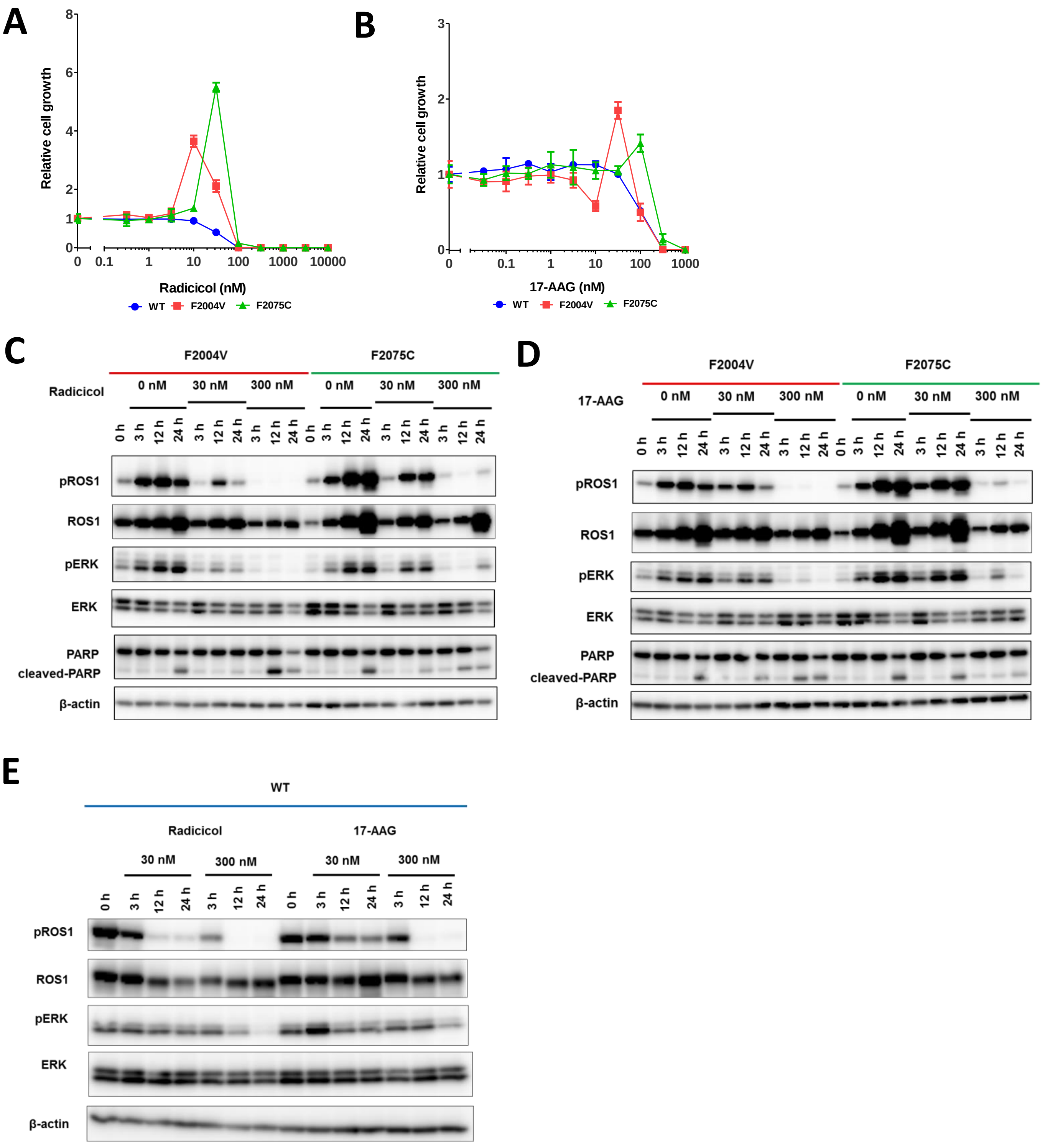

Supplementary Figure S9. Rescue effect of Hsp90 inhibitors on cells expressing wild-type, F2004V- or F2075C-mutated CD74-ROS1.
(A, B) Sensitivity to radicicol (A) or 17-AAG (B) of wild-type CD74-ROS1, F2004V- or F2075C-mutated cells. Each cell line was treated with the indicated dose ranges of inhibitors for 72 h, then cell viability was measured by the CellTiter-Glo assay. Each value was normalised with the value from untreated cells. (C-E) Immunoblots of F2004V- (C), F2075C (D)-mutated cells or wild-type CD74-ROS1 (E) cells treated with radicicol or 17-AAG. Cell lysates were immunoblotted to detect the indicated proteins.

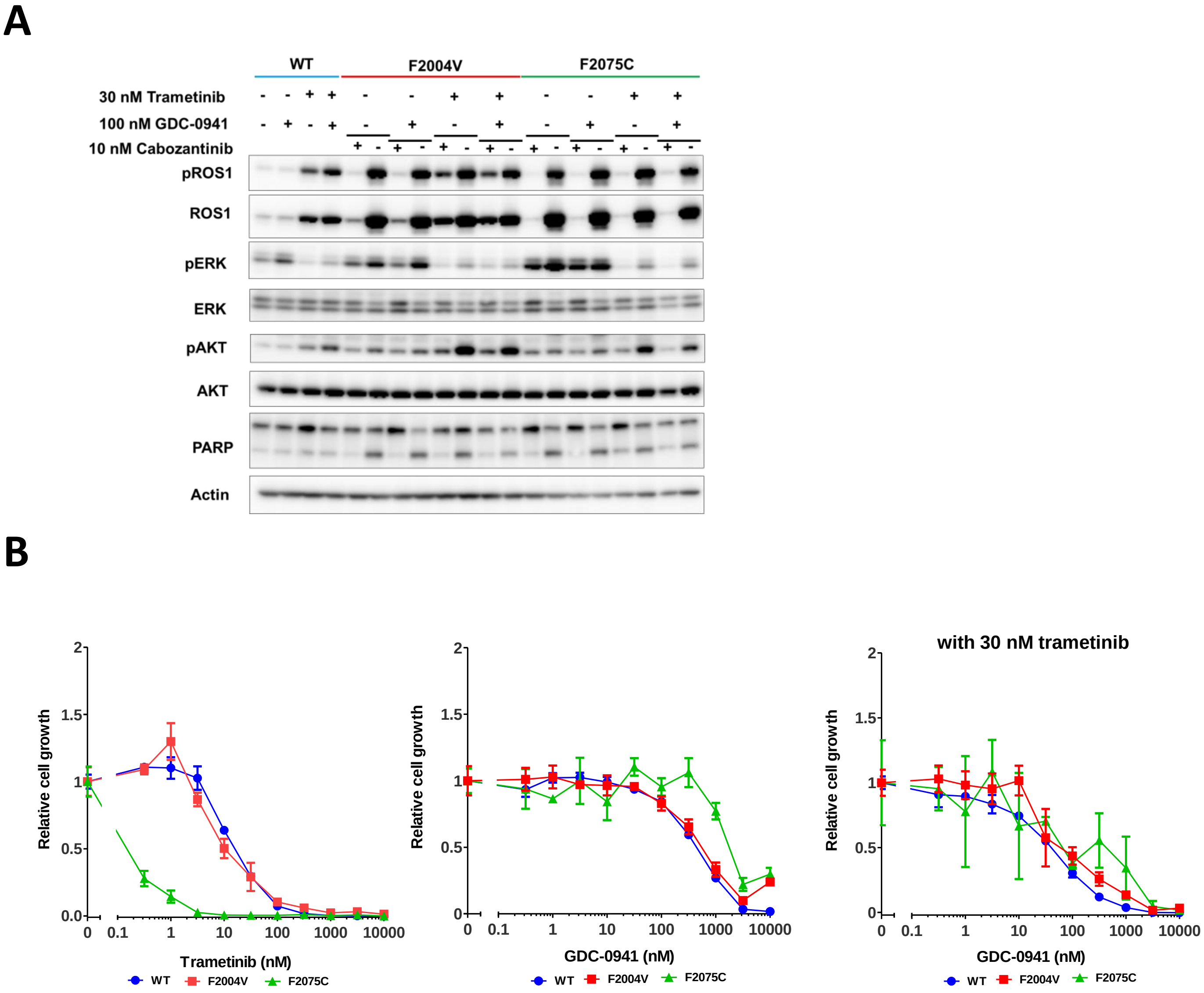

Supplementary Figure S10. Rescue effect of MEK or PI3K inhibitors on cells expressing wild-type, F2004V- or F2075C-mutated CD74-ROS1.

(A) Immunoblots of wild-type (WT) CD74-ROS1,F2004V- or F2075C-mutated cells treated with 30 nM of trametinib or 100 nM of GDC-0941 with or without 10 nM of cabozantinib for 24 h. Cell lysates were immunoblotted to detect the indicated proteins. (B) Sensitivity to trametinib (B, left), GDC-0941 (B, middle), or GDC-0941 with 30 nM of trametinib (B, right) of wild-type CD74-ROS1, F2004V- or F2075C-mutated cells. Each cell line was treated with the indicated dose ranges of inhibitors for 72 h, then cell viability was measured by the CellTiter-Glo assay. Each value was normalised with the value from untreated cells.

Figure 2A

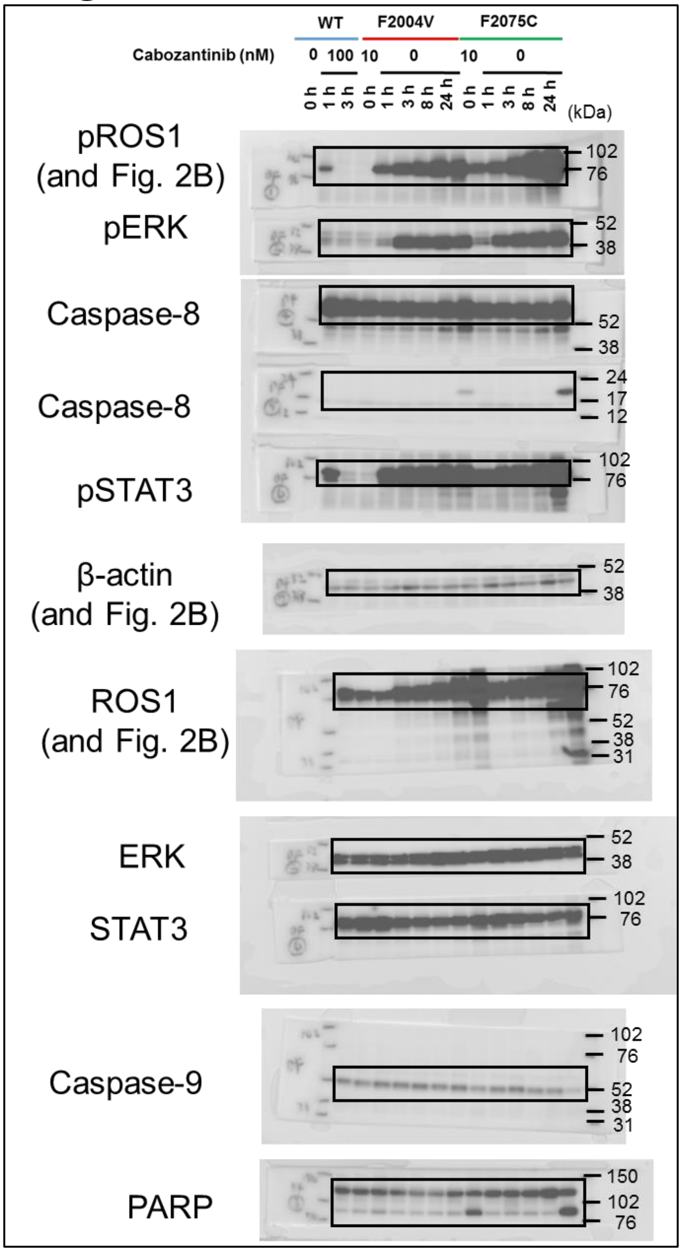

Figure 2B

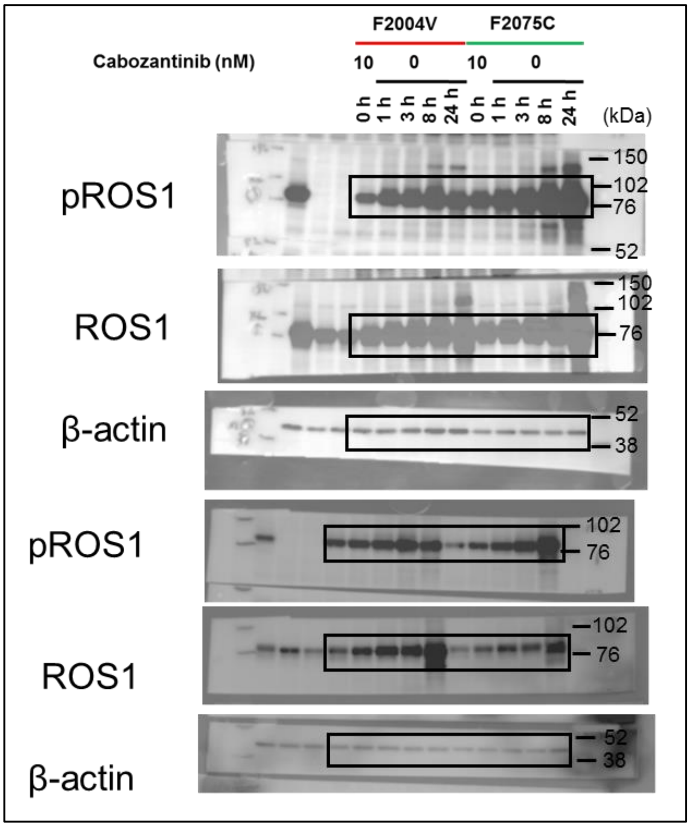

Supplementary Figure S11. Uncropped gel or immunoblot images for indicated figures

Figure 2C

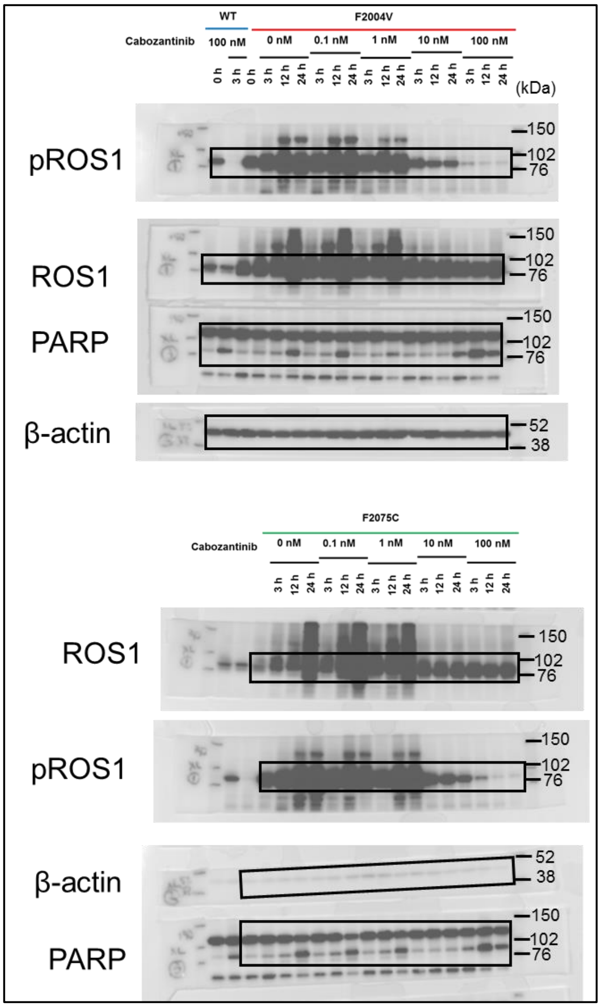

Figure 3A

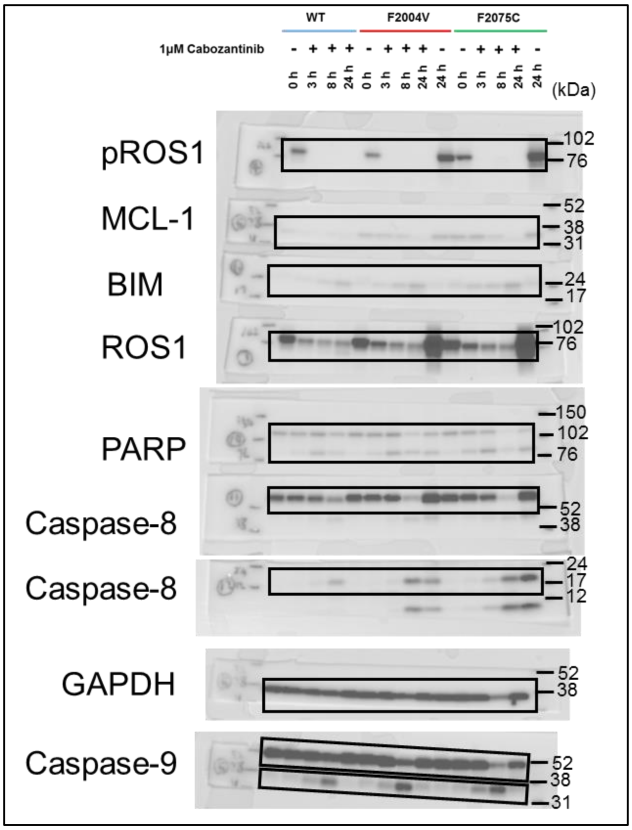

Figure 5D

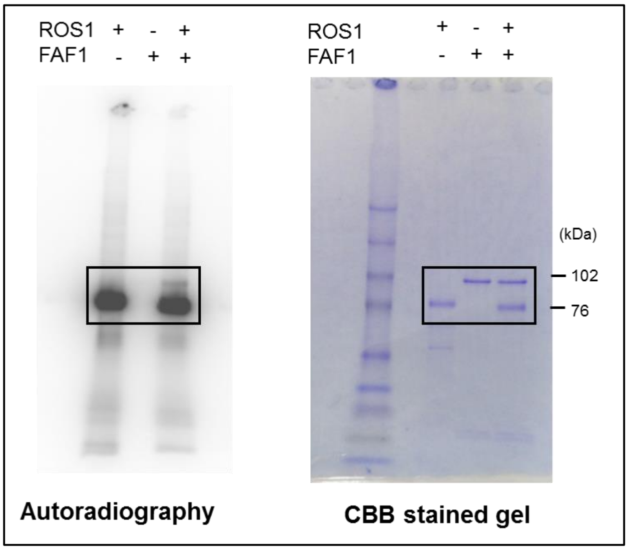

Supplementary Figure S11. Continued

Figure 7C

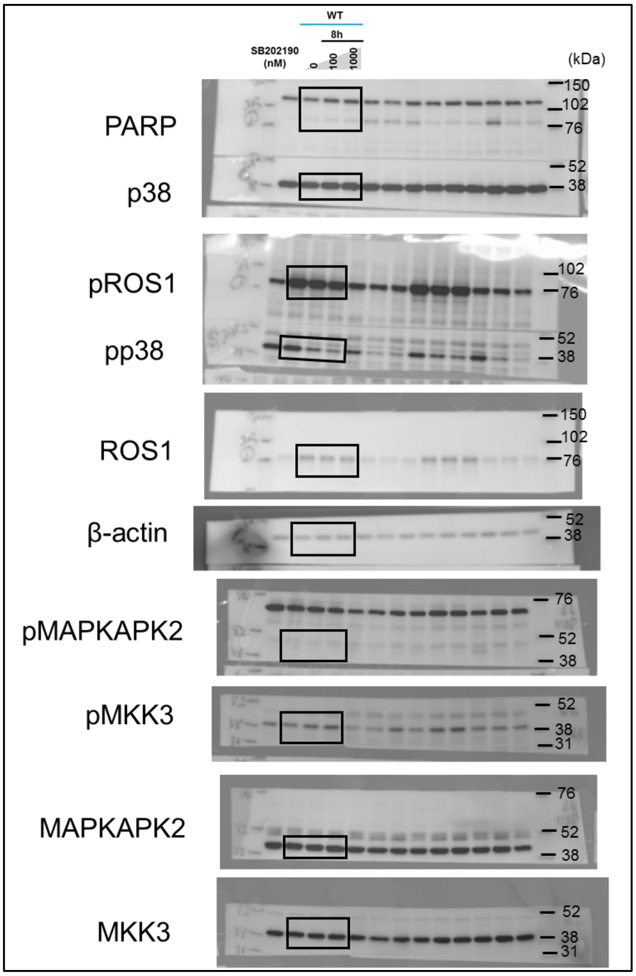

Figure 7C

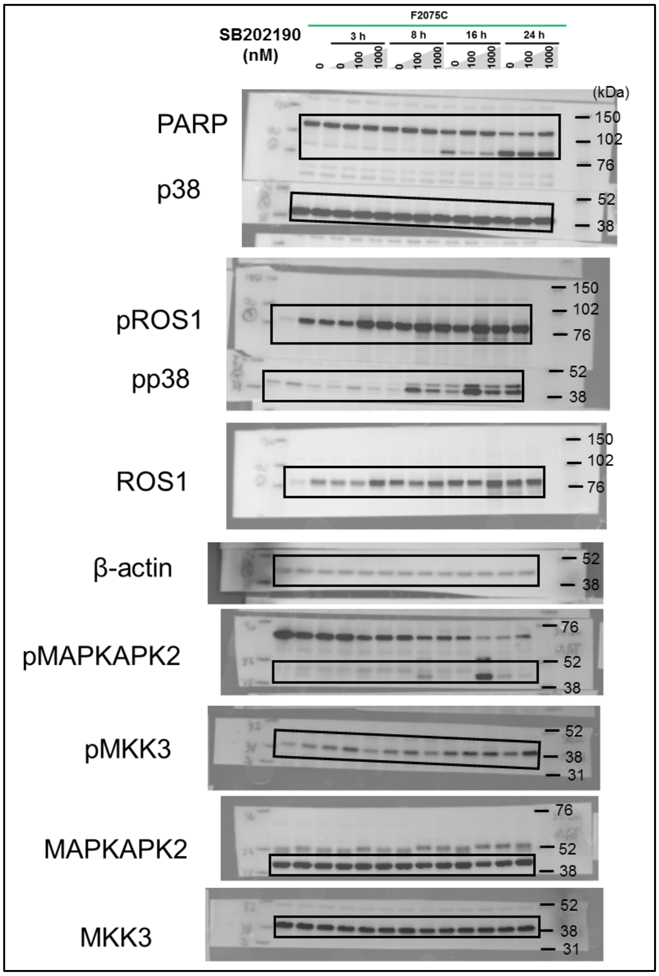

Supplementary Figure S11. Continued

Figure 7D

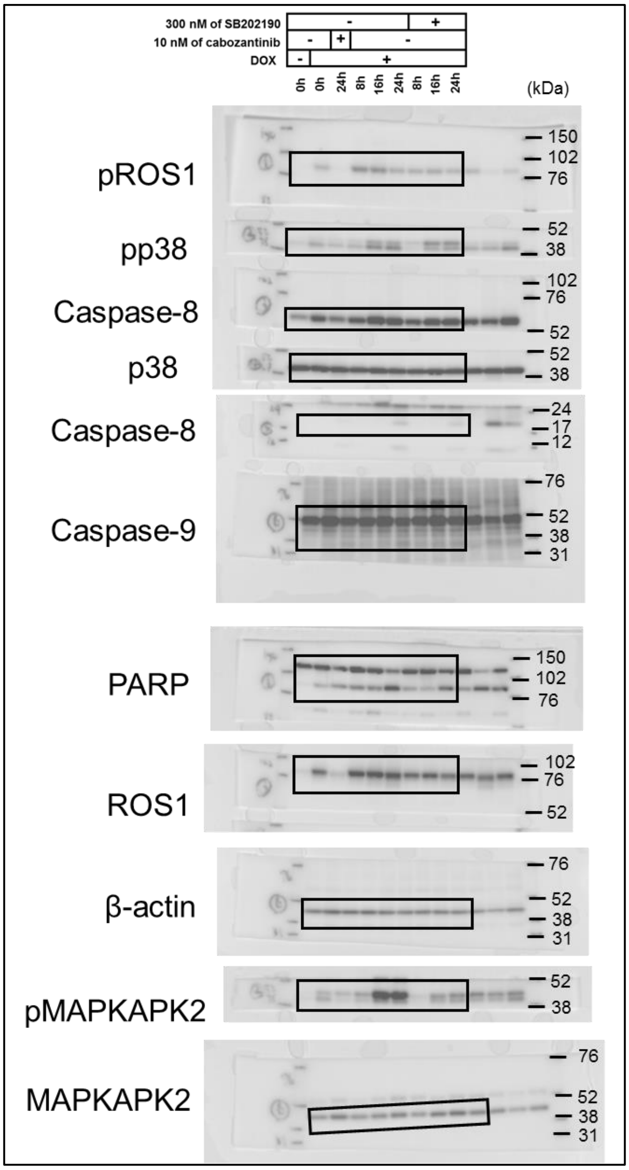

Supplementary Figure S11. Continued

A

	WT_1h (Untreated / 100 nM cabozantinib)		F2004V_1h (Cabozantinib removal /10 nM cabozantinib)		F2004V_8h (Cabozantinib removal /10 nM cabozantinib)		F2075C_1h (Cabozantinib removal /10 nM cabozantinib)		F2075C_8h (Cabozantinib removal /10 nM cabozantinib)	
	count	%	count	%	count	%	count	%	count	%
pS	3504	87.2	3519	87.3	3520	87.3	3514	87.2	3511	87.3
pT	407	10.1	406	10.1	406	10.1	406	10.1	406	10.1
pY	108	2.7	107	2.7	107	2.7	108	2.7	107	2.7
Total	4019	100	4032	100	4033	100	4028	100	4024	100

B

		WT_1h		F2004V_1h		F2004V_8h		F2075C_1h		F2075C_8h	
		count	%	count	%	count	%	count	%	count	%
DOWN < mean - 2SD	pS	95	91.3	83	85.6	29	76.3	72	87.8	29	85.3
	pT	5	4.8	9	9.3	9	23.7	10	12.2	5	14.7
	pY	4	3.8	5	5.2	0	0.0	0	0.0	0	0.0
	Total	104	100	97	100	38	100	82	100	34	100
UP > mean + 2SD	pS	111	89.5	99	86.1	56	42.1	79	66.4	66	46.2
	pT	8	6.5	10	8.7	3	2.3	7	5.9	6	4.2
	pY	5	4.0	6	5.2	74	55.6	33	27.7	71	49.7
	Total	124	100	115	100	133	100	119	100	143	100

Supplementary Table S1. Phosphopeptides identified by phosphoproteomic analysis of wild-type CD74-ROS1, F2004V- or F2075C-mutated cells under various cabozantinib treatment conditions.
(A, B) Each value is shown as mean of two independent experiments . (A) Phosphorylation sites and distribution of each amino acid on phosphoproteomic analysis. (B) Significantly upregulated (>mean + 2 SD) and downregulated (<mean –2 SD) phosphorylation sites and distribution of each amino acid on phosphoproteomic analysis.
